# Mode of Action of AlgE1: A Modular Mannuronate C‑5 Epimerase

**DOI:** 10.1021/acs.biochem.5c00156

**Published:** 2025-06-23

**Authors:** Agnes B. Petersen, Anita Solem, Gerd Inger Sætrom, Håvard Sletta, Mirjam Czjzek, Finn L. Aachmann, Anne Tøndervik

**Affiliations:** † Norwegian Biopolymer Laboratory (NOBIPOL), Department of Biotechnology and Food Science, NTNU Norwegian University of Science and Technology, Trondheim 7034, Norway; ‡ Department of Biotechnology and Nanomedicine, SINTEF Industry, Trondheim 7034, Norway; § Station Biologique de Roscoff, Sorbonne Université, CNRS, 56427 Laboratoire de Biologie Intégrative des Modèles Marins LBI2M, Roscoff 29680, France

## Abstract

The mannuronate C-5 epimerase AlgE1 from introduces long blocks of guluronate (G) into alginate. AlgE1 is an elongated enzyme consisting of six modules, of which two are catalytically active modules (A-modules). For industrial applications, G-rich alginates are sought after, and previous studies have shown that AlgE1 can be used for the valorization of both seaweed-derived and microbially produced alginates, but a complete understanding of the mode of action of AlgE1 is lacking. This study gives new data on the overall shape and conformational freedom of the AlgE1 enzyme in solution in the presence and absence of a substrate. With this basis, the questions of how the modules of AlgE1 work together and how the enzyme moves on the substrate have been addressed. The two A-modules were inactivated individually, which clarified the roles of each A-module and showed that small changes in the full-length construct affect the mode of action. The relative positions of the A-modules were switched, which resulted in two new enzymes with an initial reaction rate higher than that of the WT but with a reduced capacity to form long G-blocks. To understand the orientation of AlgE1 in processing of its substrate, lyase activity was introduced at different positions in AlgE1, and it could be concluded that AlgE1 processes the substrate with the C-terminal acting first. Overall, this study gives a completely new insight into the mode of action of AlgE1, which is important for further development and use of alginate epimerases in industrial applications.

## Graphical abstract

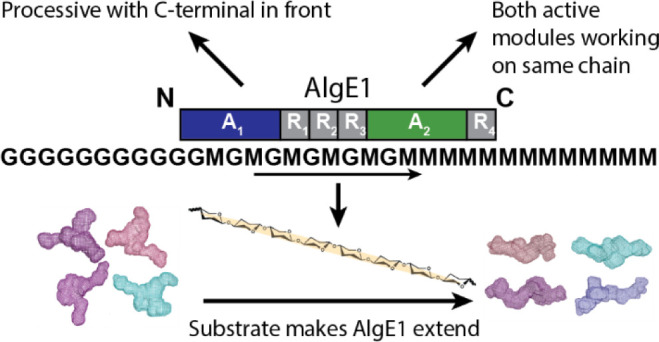

## 1 Introduction

Alginates are linear, anionic polymers of high industrial and medical importance due to their properties such as the ability to form hydrogels under physiological conditions and their biocompatibility.^1,2^ The monomeric units of alginates are β-d-mannuronic acid (M) and its C-5 epimer α-l-guluronic acid (G), linked by 1,4-glycosidic linkages^3^ (Figure 1C). The monomers can be arranged in blocks of consecutive M-residues (polyM), G-residues (polyG), or alternating M- and G-residues (polyMG), or with a more random arrangement.^4,5^ The arrangement of M- and G-residues and especially the proportion of G-residues and G-blocks are important for the structure and properties of alginates.^6^ Consecutive M-residues are rotated 180° relative to each other, forming a flexible polymer with a dimer length of 10.4 Å, while G-residues take up a different conformation with a dimer length of 8.7 Å, resulting in consecutive G-residues forming a more rigid and compact polymer.^7^ G-blocks of four residues or longer can bind divalent cations such as Ca^2+^ which is described by the so-called egg-box model,^8,9^ while M-blocks do not have the same chelating ability.^1,10^ The binding of divalent metal ions results in hydrogel formation, which is one of the reasons for the wide application of G-rich alginates in the food and pharmaceutical industries.^11^

**Figure 1:**
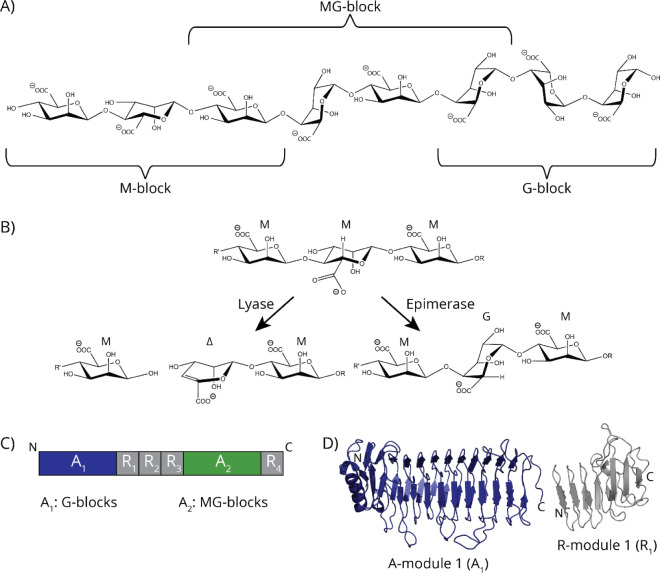
Overview over AlgE1 and alginate block structures. A) Structure of alginate oligomers showing an M-block, an MG-block, and a G-block^3^. B) Lyase and epimerase reactions were exemplified with polyM. C) Schematic structure of AlgE1 with catalytically active A-modules highlighted in blue and green. Previous studies show that when expressed individually, A_1_ creates G-blocks and A_2_ creates MG-blocks^28^. D) Structure of A_1_ and R_1_ created using AlphaFold 3 ^27^ and color-coordinated with the scheme in (C).

Alginates for commercial applications are extracted from brown seaweeds, but alginates are also produced by bacterial species of the genera and .^12,13^ Alginates are synthesized as polyM, and G-residues are subsequently introduced by an enzymatic reaction carried out by mannuronate C-5 epimerases (alginate epimerases).^11,14^ Knowledge on this class of enzymes is mostly obtained by studies of bacteria, but genes encoding alginate epimerases have also been identified in the genomes of several brown algae.^15^ Yet only two algal epimerases have been expressed and characterized.^16–19^

Bacterial alginate epimerases are categorized as AlgG and AlgE types.^11^ AlgG epimerases are located in the periplasmic space and introduce single G-residues in polyM during the translocation of alginate out of the bacterial cell.^20^ AlgE epimerases are extracellular Ca^2+^-dependent enzymes that can create longer MG or GG motifs in alginate.^21,22^ All bacteria that produce alginate encode one AlgG enzyme,^23^ whereas AlgE-type epimerases have been identified in three bacteria: , , and .^21,24–26^

AlgE-type epimerases consist of one or two A-modules with catalytic activity and one to seven R-modules that function as carbohydrate-binding modules.^21,24,28,29^ The A-modules have different substrate specificities and create different alginate structures, and by having different AlgEs with different combinations of A and R modules, bacteria can create a wide variety of alginate structures.^11^ AlgEs act in a processive manner on the alginate substrate by binding and performing several epimerization reactions, creating blocks of MG or GG before detaching from the substrate.^30–33^ produces seven AlgE-type alginate epimerases, AlgE1–7.^21,24^ AlgE7 is the only epimerase from with pronounced dual activity, functioning both as an epimerase and a lyase capable of cleaving alginate.^34,35^

Lyases utilize a β-elimination mechanism to break glycosidic linkages in the alginate chain, thereby degrading alginate to oligo- or monosaccharides. The β-elimination mechanism results in the formation of a 4,5-unsaturated moiety, 4-*deoxy*-l-*erythro*-hex-4-enopyranosyluronate (Δ), at the nonreducing end of the polysaccharide or the release of a Δ monomer.^36,37^ The alginate epimerases and lyases share a similar three-step reaction mechanism, only differing in the last step.^11,38^ In the first step, the charge of the carboxylate is neutralized. In the second step, a proton is abstracted from C-5. In the third step of the epimerase reaction, a proton is donated to C-5 from the opposite side of the ring, resulting in the formation of the G epimer of M. In the third step of the lyase reaction, the glycosidic bond is broken, and a double bond is formed between C-4 and C-5.

AlgE1 is active on polyM and introduces very long G-blocks, as well as some MG-blocks.^39^ AlgE1 consists of two A-modules, where the MG-block formation is attributed to the C-terminal A-module (A_2_), while the G-block formation is attributed to the N-terminal A-module (A_1_) (Figure 1A,B)^29^. A_1_ is followed by three R-modules (R_1_–R_3_), and A_2_ is followed by one R-module (R_4_) (Figure 1A,B)^21^. The presence of one or more R-modules increases the activity of the A-modules, compared to having the A-modules alone.^29^ AlgE1 is active on polyM and much less active on polyMG, and it seems to prefer substrates with preexisting G-blocks^28,33^ . AlgE1 introduces long G-blocks to polyM, making AlgE1 useful for the production of high G-content alginate.^40^ Because of this, AlgE1 can be used to increase the G-content in alginates extracted from seaweeds, thereby enabling valorization of low G-content alginates.^41,42^

Previous studies have reported on the activity of the A-modules of AlgE1, when the enzyme was expressed in two separate parts, where AlgE1–1 and AlgE1–2 consisted of the modules A_1_R_1_R_2_R_3_ and A_2_R_4_, respectively.^28^ This showed that AlgE1–1 extends existing G-blocks and introduces G-residues into MG-blocks and AlgE1–2 introduces G-residues in an alternating manner, producing MG-blocks from M-blocks^28^. Although A_1_ can epimerize polyM independent of A_2_, its activity is several hundred times lower than the activity of the native enzyme.^29^ In the study by Ertesvåg et al.,^28^ mixing together equimolar amounts of AlgE1–1 and AlgE1–2 restored the ability to form G-blocks, but the amount of consecutive G-residues formed was lower than that of the AlgE1 WT, maybe indicating that the mode of action of AlgE1–1 + AlgE1–2 is different from that of the WT. Furthermore, the study found indications that the catalytic efficiency of the two separate halves of the enzyme could be different from their efficiency in the native construct. While this study informs us about the activity of the A-modules separately, the effect of all modules of the enzyme together and the possible interplay between them are not clearly understood.

Despite being the subject of several studies, the complexity of AlgE1 renders elucidating its mode of action challenging. Increased knowledge of how AlgE1 works would not only open possibilities for more accurate design of alginate-active enzymes but also increase the understanding of the structure–function relationship in alginate epimerases in general and specifically for the enzymes with two catalytic domains. Furthermore, it would also increase our understanding of processive reactions on polysaccharides, in general. In this work, we therefore sought to further clarify the roles of the two catalytically active modules in AlgE1 and how they work in relation to each other. The hypothesis of the study is that the A-modules of AlgE1 work collaboratively on the same alginate chain and that AlgE1 processes with the C-terminal first. After exploring the overall structure of AlgE1 in solution, this hypothesis is then investigated through three approaches: Through inactivating the catalytic domains, the activity of each active site was characterized and the importance of the interplay between the modules was confirmed. Switching the relative positions of the A-modules revealed that the modules work in a collaborative manner on the same substrate chain. Lastly, the insertion of the AlgE7 A-module and thus the introduction of lyase activity to the enzyme showed that AlgE1 processes with the C-terminal first.

## 2 Materials and Methods

### 2.1 Alginate Substrates

PolyM was produced using an AlgG-deficient strain of resulting in polyM with *F*_M_ = 1.0.^43^ For NMR analysis, polyM was acid-hydrolyzed to a degree of polymerization (DP) of 70–100.^44^ ^13^C-1-labeled substrate was produced from the same strain using ^13^C-1-labeled D-fructose as a carbon source. After purification, the ^13^C-1-labeled polyM was depolymerized using the same method as the unlabeled polyM.

PolyMG was produced by epimerization of polyM using the AlgE4 epimerase until the reaction was complete,^45^ thereby obtaining a final product with *F*_G_ = 0.46 and *F*_GG_ = 0.

### 2.2 Mutagenesis, Protein Production, and Purification

#### 2.2.1 Construction of Expression Plasmids

Plasmids for the expression and purification of AlgE1 with combinations of active and inactive A-modules (Table 1) were constructed by ligation of synthetic gene fragments (obtained from GenScript) into pTYB1. First, NdeI-NotI fragments (2707 bp) containing the A_1_-module with either Y149 (WT) or F149 (inactive) as well as R1R2R3 were inserted into pTYB1. Then, NotI-XhoI fragments (1513 bp) containing the A_2_-module with either Y993 (WT) or F993 (inactive) and R4 were inserted downstream. This sequential cloning strategy resulted in the addition of a three-amino-acid motif (GGR) at the ligation site between the two fragments.

**Table 1:**
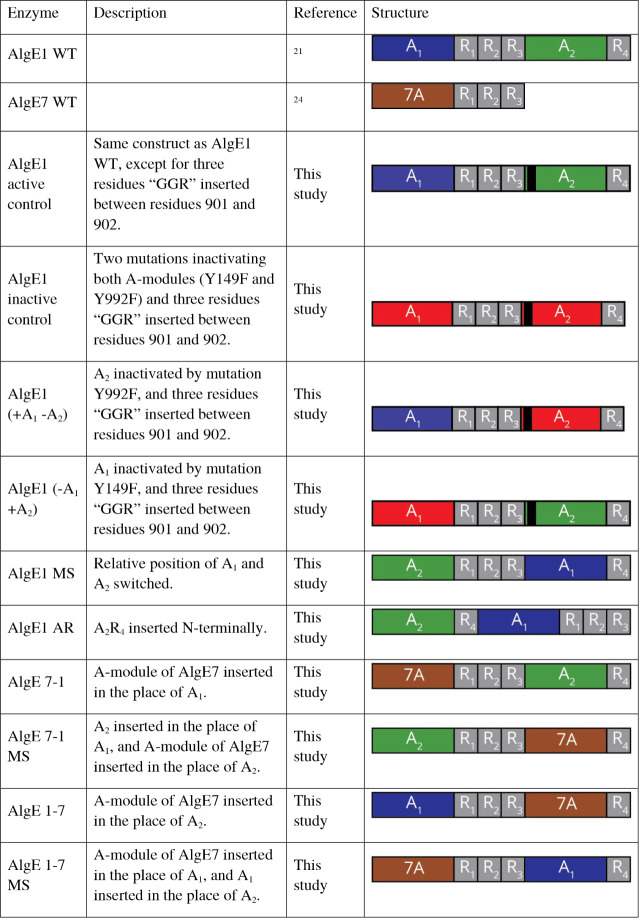
Plasmids Used in This Study

Plasmids for the expression and purification of AlgE1 MS and AlgE1 AR were constructed by the insertion of synthetic NdeI-XhoI gene fragments (obtained from GenScript) into pTYB1.

Two new chimeric enzymes were constructed based on AlgE1 with the A-module inserted instead of either A_1_ (AlgE 7–1) or A_2_ (AlgE 1–7). The construction of AlgE 7–1 was done by restrictiing endonuclease digestion, followed by T4 DNA ligation. A 1020 5′ NdeI-PfoI fragment of *AlgE1* was replaced by the corresponding 1020 5′ NdeI-PfoI fragment of *AlgE7*. AlgE 1–7 was constructed by replacing 1131 bp corresponding to AlgE1 A_2_ with a 1131 bp PCR-amplified fragment of *AlgE7* A in a Gibson Assembly reaction using DNA HiFi MasterMix (New England Biolabs) and following the protocol of the manufacturer.^46^ The relative positions of the A-modules in AlgE 7–1 and AlgE 1–7 were changed to produce AlgE 7–1 MS and AlgE 1–7 MS. AlgE 7–1 MS was constructed in a Gibson assembly reaction, as described above. AlgE 1–7 was constructed by first exchanging A_2_ with AlgE1 A_1_ by Gibson Assembly, followed by replacing the 1020 5′ NdeI-PfoI fragment of the resulting construct with the 1020 5′ NdeI-PfoI fragment of *AlgE7*. For the construction of AlgE 7–1 MS, AA 6 to AA 376 was exchanged with AA 850 to AA 1220. For the construction of AlgE 1–7 MS, AA 847 to AA 1222 was exchanged with AA 4 to AA 379.

PCR was performed on a C1000 Touch Thermal Cycler (Bio-Rad) using Q5 High-Fidelity DNA Polymerase (New England Biolabs). The primers used for PCR amplifications are listed in Table S1. PCR products were purified using the Zymo DNA Clean and Concentrator kit (Zymo Research).

Construct sequences were verified by Sanger sequencing, performed by Eurofins Genomics. The constructed AlgE 7–1 and AlgE 1–7 were inserted into the expression vector pTYB1 (New England Biolabs) as NdeI-*Eco*RI fragments. In this vector, the enzymes are under the control of the T7/lac promoter system.

Construction of enzymes was performed using DH5α (Bethesda Research Laboratories) as host. was grown at 37 °C in Luria Broth (LB) (10 g/L tryptone, 5 g/L yeast extract, and 5 g/L NaCl). For selection, the growth medium was supplemented with 100 μg/mL ampicillin. For growth on solid media, 15 g/L of bacteriological agar was supplemented to the liquid growth medium. Transformation of bacteria was done according to an RbCl transformation protocol.^47^ Plasmids were extracted and purified using a Wizard Plus SV Miniprep Kit (Promega). DNA extraction from agarose gels was done by using Zymoclean Gel DNA Recovery Kits (Zymo Research).

#### 2.2.2 Expression and Purification

Enzyme production and purification have previously been characterized in depth.^24,28,29^ Enzymes were expressed in ER2566 (New England Biolabs). Expression was induced by the addition of 0.5 mM isopropyl β-d-1-thiogalactopyranoside (IPTG) to cultures grown to OD = 0.8–1.0 in 2xLB. Expression was continued for 16–20 h at 16 °C before the cells were harvested by centrifugation (5500 RCF, 4 °C, 5 min). Cells were lysed by sonication at 40% amplitude for a total of 3.5 min, in 30 mL lysis buffer (20 mM HEPES, 5 mM CaCl_2_, 500 mM NaCl, 0.05% Triton X-100, pH 6.9). The resulting suspension was centrifuged (20 000 RCF, 4 °C, 30 min), and the supernatant was filtered through a 0.22 μm cellulose-nitrate membrane filter. The filtered solution was loaded onto an XK 16/20 column (Cytiva) packed with 20 mL of chitin beads (New England Biolabs). The proteins were purified on a KTA Pure FPLC (Cytiva). Before loading the protein, the column was equilibrated by running 3x column volume (CV) of washing buffer (20 mM HEPES, pH 6.9, 5 mM CaCl_2_, 500 mM NaCl) through the column, and after loading, the protein was washed with 10xCV of the same buffer at 5 °C. The protein was cleaved off the column by washing with 3xCV cleavage buffer (20 mM HEPES, pH 6.9, 5 mM CaCl_2_, 500 mM NaCl, 50 mM DTT) at 5 °C and incubating for 16–40 h at room temperature. Afterward, the protein was eluted with the washing buffer. Fractions containing the desired purified protein, as detected by gel electrophoresis, were collected and dialyzed against dialysis buffer (5 mM HEPES, pH 6.9, 5 mM CaCl_2_) before freeze-drying. Freeze-dried proteins were stored at −20 °C and dissolved in the appropriate solvent for analysis. Protein purity was assessed using an SDS-PAGE analysis (SurePAGE Bis-Tris 12% gel (GenScript) with Precision Plus Dual Color standard (Bio-Rad), and stained using an eStain L1 Protein Staining Device (GenScript)), and concentrations were determined on a NanoDrop One spectrophotometer (Thermo Fischer Scientific) using ε = 110 035 M^–1^ cm^–1^ calculated using the ExPASy ProtParam online tool.^48^

### 2.3 SEC-SAXS Experiments

SAXS data of AlgE1 alone and in the presence of oligoM DP20 were collected at the Synchrotron SOLEIL on the SWING beamline (details shown in Table S2). Protein samples were centrifuged prior to analyses to remove potential aggregates. SAXS measurements were coupled with prefixed size exclusion chromatography (SEC). For AlgE1, the SEC column (Agilent Biosec-5, 500 Å) was equilibrated in buffer C (75 mM NaCl, 5 mM CaCl_2_, 20 mM HEPES, pH 6.9), the same as that used for protein purification. To analyze a sample of AlgE1 in the presence of oligoM DP20, 50 mL of buffer C with 9.3 mg of oligoM DP20 was prepared, which was used for SEC column equilibration. oligoM DP20 (0.04 mg) was added to 200 μL of the AlgE1 (7.0 mg/mL) sample solution 30 min prior to measurement. After calibration of the SEC column, the AlgE1 + oligoM DP20 solution was injected. For data collection of both AlgE1 and AlgE1 + oligoM DP20, 70 μL of the protein sample was injected onto the Bio SEC column, and, triggered by the elution procedure, a first series of 180 successive frames of 750 ms were recorded on the buffer solution (before the column’s void volume) to measure the background. In the next step, 250 frames were collected continuously during the elution with a frame duration of 1.5 s and a dead time between frames of 0.5 s (elution at 0.2 mL/min). The sample–detector distance was 2.4 m, resulting in a scattering vector *q*-range of 0.012–0.504 Å^–1^ for both samples. The obtained scattering data were normalized and corrected according to standard procedures using FOXTROT (SOLEIL). Following inspection of the *R*_g_ value for individual frames as a function of the elution profile, frames with constant *R*_g_ values ranging from 380 to 420 for AlgE1 and 340 to 405 for AlgE1 + oligoM DP20 were averaged. The Guinier equation was used to calculate the forward scattering *I*(0) and the radius of gyration *R*_g_. The distance distribution function *P*(*r*) and the maximum particle dimension *D*_max_ were calculated by Fourier inversion of the scattering intensity *I*(*q*) using GNOM, as integrated in the PRIMUS software (ATSAS 3.1.3).^49^ Models of protein envelopes were calculated from the experimental scattering curves using DAMMif (37).^50^ The global fit parameter χ^2^ is used for evaluating the discrepancy between the computed scattering profile from a model and the measured scattering profile, and the smaller the number, the greater the confidence in the model.^51^ χ^2^ is defined as follows:^51^

$$\chi^{2}=\frac{1}{N-1}\overset{N}{\underset{j=1}{\sum }}{[\frac{I_{\exp}\left(q_{j}\right)-cI_{\mathrm{mod}}\left(q_{j}\right)}{\sigma \left(q_{j}\right)}]}^{2}$$

where *N* is the number of points in the scattering profile, *I*_exp_(*q*) is the experimental scattering profile, *c* is a multiplicative scaling parameter that is used to minimize χ^2^, *I*_mod_(*q*) is the computed scattering profile based on the three-dimensional model, and σ­(*q*) is the standard error for each measured data point.^51^ Relative χ^2^ values are most valuable for comparing two models against the same dataset, while absolute values are less useful.^51^

Variable χ^2^ values, especially for AlgE1 in the presence of oligoM DP20, possibly indicated a mixture of conformers. Therefore, the data were analyzed using EOM.^52,53^ For this, rigid bodies and linkers were defined based on the structure predicted by the AlphaFold3 server.^27,54,55^ EOM was used to calculate a quantitative measure of flexibility, *R*_flex_, based on the distributions of *R*_G_ and *D*_max_, and *R*_σ_ was calculated based on the standard deviations for the distributions of the selected ensemble and the pool.^53^ Experimental and processed data were visualized in SASPLOT as part of PRIMUS and exported to Excel to produce the graphics. Figures of models and envelopes were created using the PyMOL Molecular Graphics System v2.5.4 (Schrödinger, LLC).

### 2.4 Activity Assays

Absorption-based activity assays were performed in 96-well UV plates (Corning) using polyM, polyMG, or polyG (1 mg/mL, buffer: MOPS 10 mM, pH 6.9, NaCl 100 mM, CaCl_2_ 2.5 mM for inactivated mutants, or buffer: HEPES 10 mM, pH 6.9, NaCl 75 mM, CaCl_2_ for AlgE1-AlgE7 chimeras), adding alginate epimerase (0.5 μM (inactivated mutants) or 0.3 μM (AlgE1-AlgE7 chimeras)) to start the reaction and then measuring absorbance at 230 nm every 5 min for 5–5.5 h at 25 °C with 5 s of shaking before each measurement (Infinite M200 (Tecan), CLARIOstar Plus, or PHERAstar FSX (BMG Labtech)). To enable observing the formation of MG and GG motifs by the epimerases, after 2.5 h, a G- or GG-specific lyase was added and then continuously measured for 2.5 h afterward.^56,57^ All measurements were performed in triplicates (*n* = 3).

### 2.5 NMR Analysis

#### 2.5.1 Time-Resolved NMR

Before starting a time-resolved experiment, a 1D ^1^H or ^13^C spectrum was recorded for the substrate. Then, a pseudo-2D experiment was obtained by recording a ^1^H or ^13^C 1D spectrum every 5 min for 14–16 h at 25 °C. Afterward, a ^1^H–^13^C HSQC was recorded for product characterization. ^1^H signals were internally referenced to the water signal, and ^13^C signals were indirectly referenced to the water signal based on absolute frequency ratios.^58^ All time-resolved NMR spectra were recorded on a Bruker Avance III HD 800 MHz spectrometer using a 5 mm Z-gradient CP-TCI (H, C, N) cryogenic probe.

#### 2.5.2 NMR Measurements

To characterize the total level of epimerization, an epimerization reaction was performed with enzyme (25 μg/mL) and polyM (2.5 mg/mL, DP 70–100) or oligoMG (2.5 mg/mL, DP 80) in a buffer containing 10 mM MOPS (pH 6.9), 100 mM NaCl, and 2.5 mM CaCl_2_. The reactions were performed in triplicates at room temperature at 200 rpm for 48 h. Afterward, ethylenediaminetetraacetic acid (EDTA) (5 mM) was added to the reaction mixtures, and the reactions were stopped by boiling for 90 °C for 15 min. The reaction mixtures were freeze-dried and redissolved in 600 μL of D_2_O (99.9%, Sigma-Aldrich) with 0.05% 3-(trimethylsilyl)-propionic-2,2,3,3-d_4_ acid sodium salt (TSP) as an internal reference. A 1D ^1^H NMR spectrum of each sample was recorded at 83 °C with 64 scans and a spectral width of 10 ppm. All NMR spectra recorded at 83 °C were recorded on a 400 or 600 MHz BRUKER NEO instrument (Bruker BioSpin AG, Fällanden, Switzerland) equipped with a 5 mm iProbe TBO preheated to 83 °C.

Key signals were integrated, and the relative amounts of different residue motifs were calculated based on the previously published approach.^44^

All NMR magnets were located at the NV-NMR Center at the Norwegian University of Science and Technology (NTNU). All spectra were recorded using TopSpin 3.6 pl7 or 4.0.8 (Bruker BioSpin) and were processed and analyzed using TopSpin 4.4.0 software (Bruker BioSpin).

### 2.6 HPAEC-PAD

High-performance anionic exchange chromatography coupled with pulsed amperometric detection (HPAEC-PAD) was used for reaction and product characterization using oligomeric standards created by the degradation of polyM and polyMG using an M-lyase from as previously described.^57,59,60^

HPAEC-PAD analysis was performed on reactions with polyM and AlgE7, AlgE 7–1, and AlgE 1–7, both as an analysis at the end of the reaction and in a time-resolved manner. The endpoint HPAEC-PAD analysis was performed of samples that had previously been used for time-resolved NMR, to which ultrapure water (UPW) was added to dilute the samples to a final polysaccharide concentration of 0.3 mg/mL.

The time-resolved analysis was made of AlgE 7–1 and AlgE 1–7, where a reaction between polyM (1.0 mg/mL) and the enzymes (0.5 μM) was run for up to 24 h in a 96-well plate. At time points 0, 15, 30 min, 1, 2.5, 5, 10, and 24 h, three samples were taken from the plate wells. The reaction was stopped by boiling the samples for 10 min at 95 °C. Then, the samples were diluted with UPW to a polysaccharide concentration of 0.25 mg/mL.

All samples were analyzed on an ICS 5000+ system (Thermo Scientific) with a 4 × 250 mm IonPac AS4A main column and a 4 × 5 mm AG4A guard column. Data were collected and processed using Chromeleon 7.2 software (Thermo Scientific).

## 3 Results and Discussion

### 3.1 Small-Angle X-ray Scattering Revealed Limited Intermodular Flexibility Allowing Proximity of AlgE1 Domains for Concerted and Processive Action

To explore the relative spatial arrangement of AlgE1 domains in solution, we analyzed AlgE1 in the absence and presence of the oligoM DP20 substrate by small-angle X-ray scattering (SAXS). The measurements revealed that this multidomain epimerase features a relatively compact structural organization with limited interdomain flexibility.

The values for the radius of gyration (*R*_g_) and the maximum particle distance (*D*_max_) were larger for AlgE1 with substrate than without, with *R*_G_ increasing by 8.4 Å and *D*_max_ increasing by 41 Å (Table 2). This shows that the enzyme undergoes a conformational rearrangement to a more extended form in the presence of the substrate.

**Table 2:** Summary of the EOM Analyses to Determine the Flexibility of AlgE1 in Solution^a^

	*R*_g_ [Å] final	*D*_max_ [Å] final	*R*_g_ [Å] ensemble	*D*_max_ [Å] ensemble	*R*_g_ [Å] pool	*D*_max_ [Å] pool	*R*_flex_ ensemble/pool	*R*_σ_
AlgE1	49.8 ± 0.5	177 ± 2	50.7	189.2	68.5	223.9	60%/86%	0.50
AlgE1 + DP20	58.2 ± 0.7	218 ± 3	56.0	191.4	68.5	224.2	71%/87%	0.56

a Final *R*_g_ and *D*_max_ derived from the unique models for the selected ensembles, averaged *R*_g_ and *D*_max_ of the selected ensembles from the EOM analysis and the pool, as well as *R*_flex_ and *R*_σ_ to describe the flexibility. *R*_flex_ of 100% or *R*_σ_ of 1 corresponds to a fully flexible system.

The structural arrangement of the DAMMif^50^ *ab initio* calculated envelopes of AlgE1 in the absence of substrate (Figure 2) highlights a bent, V-shaped, triangular, and relatively compact spatial organization of the different domains, which could resemble the organization proposed by AlphaFold3 (AF3).^27,54,55^ Despite the similarity, the structural coordinates of the AF3 model as a rigid body do not fit well the experimental curve, as calculated by CRYSOL (Figure S3). While the single envelope calculations by DAMMif fitted the experimental data better for AlgE1 with reasonable global fit parameter (χ^2^) values (Figure S3), the variability of envelopes could result from multiple conformers being present in solution. In the case of AlgE1 in the presence of oligoM DP20, the individual envelopes calculated by DAMMif showed more extended, linear shapes, although with higher χ^2^ values to fit the experimental curve, than for AlgE1 alone (Figures 2 and S4).

**Figure 2:**
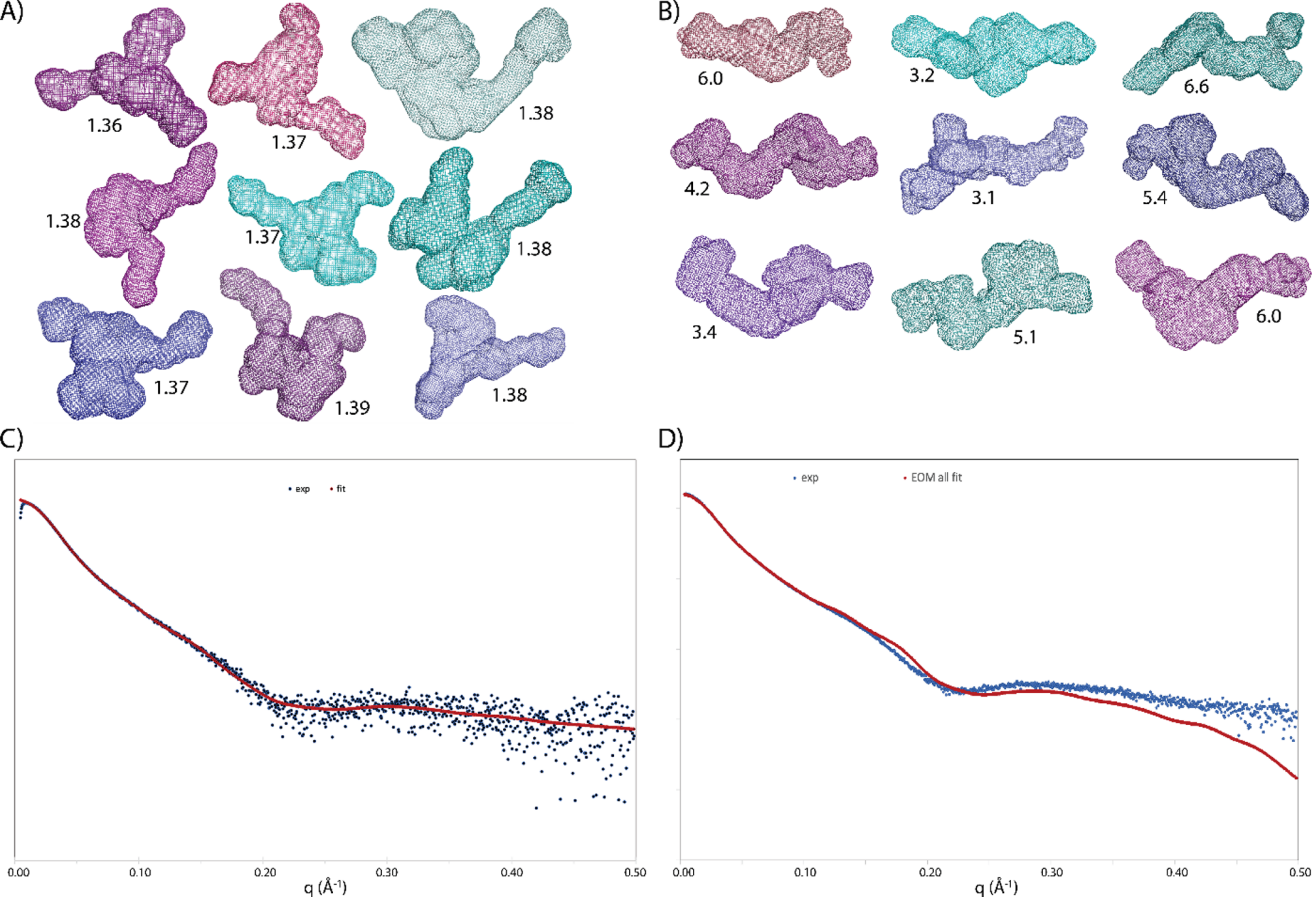
Solution SAXS curves of AlgE1 without substrate and with oligoM DP20, and structural interpretation of the experimental data. A) and B) Nine individual envelopes as calculated by DAMMif that fit the experimental data for AlgE1 without substrate (A) or AlgE1 with oligoM DP20 (B). The χ^2^ values are given for each envelope, with lower values indicating a better fit of the model. C) Best fitted curve (red line) obtained by the EOM calculation, containing 78% of the model in Figure S3B, and 11% of each of Figure S3C,D resulting in a χ^2^ value of 4.64. The blue dots represent the experimental data. D) The EOM best fit of the experimental curve of AlgE1 with oligoM DP20, containing 12% of the lime-green model, 25% of the blue-green, and 62% of the green models shown in Figure S4, resulting in a χ^2^ value of 61.62 (χ^2^ values are high because of the very low statistical errors of these experimental data).

To further analyze the possibility of conformational diversity of the enzyme in solution, we used the ensemble optimization method (EOM).^52,53^ In the EOM calculations, an ensemble is selected from a large pool of theoretical conformers, and for each of these, the corresponding theoretical scattering curve is generated. For both enzyme samples, the selected ensembles were able to fit the experimental data (Figure S5). The results confirm the presence of rather compact objects, with at least two populations of conformers (Figure 2) for both samples, which are more extended for AlgE1 in the presence of oligoM DP20. In addition, a calculated quantitative measure of flexibility (*R*_flex_) for the selected ensemble of AlgE1 alone (60%) was lower compared to that of AlgE1 in the presence of oligoM (71%) (Table 2), again pointing toward a more compact and less flexible structure of AlgE1 in solution in the absence of substrate. This is also illustrated by the respective *R*_flex_ and *R*_σ_ values (Table 2): *R*_flex_ and *R*_σ_ are values to describe the flexibility in the EOM approach, where 100% or 1 correspond to a fully flexible system, respectively.^53^ *R*_σ_ indicates the variance of the ensemble distribution with respect to the original pool.^53^

AlgE1 has a more extended conformation in the presence of substrate compared to AlgE1 alone, which is supported by the shifts to higher *R*_G_ and *D*_max_ values (Table 2) and the EOM results. Likely, this is to accommodate the oligoM (DP20: 104 Å) chain binding to the substrate-binding surface of AlgE1. The more extended and linear conformation of AlgE1 fits with a mode of action where both A-modules bind to the same substrate chain.

### 3.2 Inactivation of AlgE1 A-Modules Confirms Specificity and Shows Importance of the Collaboration between the Two A-Modules

Previous studies have described the epimerase activity of truncated versions of AlgE1 and the individual A-modules,^28,29,33^ whereas in the present study, the interplay between the two A-modules is investigated. The initial hypothesis is that the activity and processivity of each A-module within AlgE1 are influenced by the presence and activity of the other A-module.

Four variants of AlgE1 were made, where the A-modules were inactivated by a mutation of Tyr to Phe in the active sites (Y149F for A_1_ and Y992F for A_2_) (Figure 3A):

- “AlgE1 (+A_1_ −A_2_)” with A_1_ active and A_2_ inactivated
- “AlgE1 (−A_1_ +A_2_)” with A_1_ inactivated and A_2_ active
- “Active control” with both A-modules active
- “Inactive control” with both A-modules inactivated

**Figure 3:**
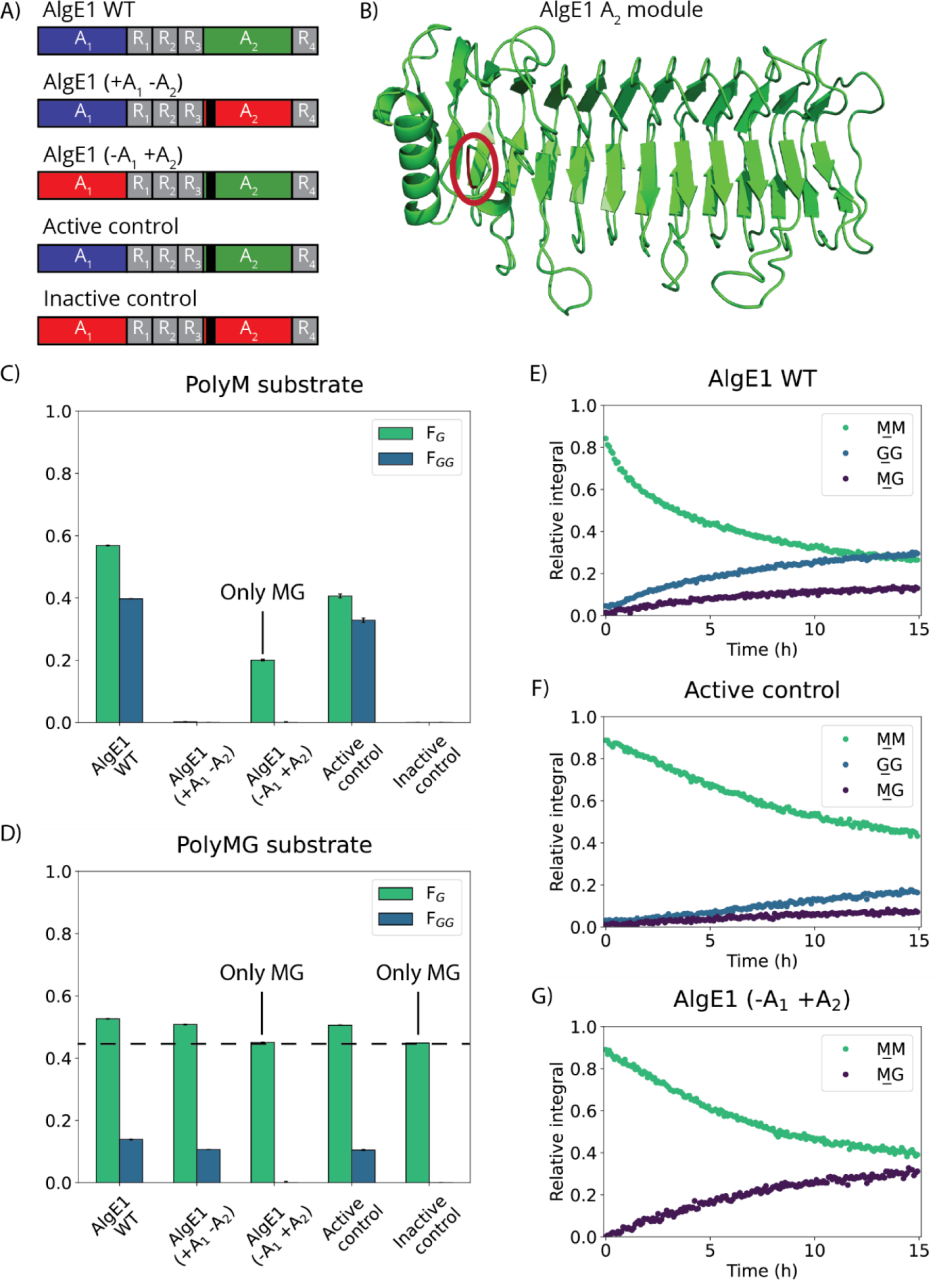
Reactions of AlgE1 mutants and AlgE1 WT with polyM or polyMG. A) Schematic overview of AlgE1 mutants compared to AlgE1 WT. B) AlphaFold 3 model of the A_2_-module with residues 901 and 902 shown in red and highlighted by a red circle. Between these two residues, the “GGR” motif was inserted. C) and D) NMR analyses of epimerization of polyM (C) or polyMG (D) after 48 h reaction. The dashed line shows the fraction of G-residues (*F*_G_) of the untreated polyMG substrate, and the labels “Only MG” show where only MG and no GG was present in the analyzed samples. All measurements are listed in Tables S3 and S4 (*n* = 3). E–G) Reaction between ^13^C-1-labeled polyM and AlgE1 WT (E), the active control (F), and AlgE1 (−A_1_ +A_2_) (G), measured using time-resolved NMR. The integral of the signal for GM has been omitted, as the signal slightly overlaps with the MM signal; thus, the integrals are not representative of the progression of the signal. The GM and MG signals both show the formation of MGMG motifs and therefore have the same progression, and so only the MG signal integrals are shown.

A three-amino-acid residue “GGR” motif was inserted at the beginning of A_2_ of all four mutants between residues 901 and 902, to allow for performing the inactivation mutation in A_2_, which means that the active control is different from the AlgE1 WT.

The activity of the four AlgE1 mutants and the AlgE1 WT on polyM was assessed using an absorbance-based activity assay. Choosing polyM as the model substrate enabled more accurate analysis of reactions performed compared to using a substrate containing both M and G. The absorbance-based activity assay showed that only AlgE1 (−A_1_ +A_2_), the active control, and AlgE1 WT were active on polyM, while AlgE1 (+A_1_ −A_2_) and the inactive control were not (Figure S6). The mode of action of the three enzymes active on polyM was investigated using time-resolved NMR (Figure 3D–F). AlgE1 (−A_1_ +A_2_) forms only MG motifs and no GG motifs, and the initial reaction rate is slower than the WTs. The active control can form a substantial amount of GG- and MG-blocks but also reacts slower than the WT. This means that the “GGR” insert must be affecting the interplay between A_1_ and A_2_ and showing that they are not working independently after each other.

To investigate the total level of epimerization from each mutant, an epimerization analysis was performed using NMR spectroscopy, where the product composition after 48 h of reaction between the enzyme and substrate in question was analyzed.^44^ The epimerization analysis was performed for each mutant and AlgE1 WT reacting with polyM. After 48 h, AlgE1 WT reached the highest fraction of G-residues (*F*_G_) = 0.57, while the active control only reached *F*_G_ = 0.41 (Figure 3B and Table S3). Both AlgE1 WT and the active control form G-blocks of 17–18 monomers. AlgE1 (−A_1_ +A_2_) was active on polyM reaching *F*_G_ = 0.20 but not forming consecutive G-residues. The experiment confirmed that AlgE1 (+A_1_ −A_2_) and the inactive control were inactive on polyM. The substrate used was a polyM with DP ∼ 70, which is a suitable substrate length for NMR analysis but too short to reach a very high degree of epimerization, as AlgE1 cannot epimerize the terminal residues of the substrate.^33^ Similarly, a reaction analysis was made with polyMG (Figure 3C and Table S4). Here, AlgE1 (−A_1_ +A_2_) and the inactive control were inactive. AlgE1 WT, AlgE1 (+A_1_ −A_2_), and the active control were active but only increasing the G-content from *F*_G_ = 0.45 to 0.51 (AlgE1 (+A_1_ −A_2_) and active control) and *F*_G_ = 0.53 (AlgE1 WT).

AlgE1 (−A_1_ +A_2_) epimerized polyM at a lower initial reaction rate than the WT, did not form G-blocks, and reached a lower *F*_G_ = 0.20. AlgE1 (−A_1_ +A_2_) was not active on polyMG. This means that the substrate for the second A-module of AlgE1 is polyM, where it forms MG motifs but cannot form G-blocks, as concluded first by the previous study.^28^ This is the same activity as when the A_2_-module is expressed alone and is in the same order of magnitude.^28^ In the active control, both modules convert M to G, and AlgE1 (−A_1_ +A_2_) reached 50% of the G-content of the active control, which means that even when A_1_ is inactivated, A_2_ can still perform reactions unhindered.

AlgE1 (+A_1_ −A_2_) was active on polyMG, but not on on polyM, where it was able to form G-blocks. The activity on polyMG and the products formed are consistent with the activity observed for the A_1_-module when only part of AlgE1 is expressed,^28^ i.e., when A_1_R_1_R_2_R_3_ alone reacted with polyMG, *F*_G_ was increased with 0.055, which is the same increase as observed in our study. Whether the A_2_-module was active or not did not affect the epimerization of polyMG, as both AlgE1 (+A_1_ −A_2_) and the active control reached the same *F*_G_ = 0.51 and *F*_GG_ = 0.10–0.11.

AlgE1 (+A_1_ −A_2_) was not active on polyM at the enzyme concentration tested first, but when the enzyme concentration was increased 5-fold, a small amount of G- and GG-product was formed (Figure S7). In previously published work, A_1_R_1_R_2_R_3_ was active on polyM forming a significant amount of G (*F*_G_ = 0.19) and G-blocks (*F*_GG_ = 0.10),^28^ which could be explained by the study using more enzyme than our study or by their substrate containing a small amount of G-residues. Possibly, the inactivation of A_2_ in AlgE1 (+A_1_ −A_2_) results in a low binding affinity of A_2_ on polyM. A study by Holtan et al. 2006 ^33^ observed high activity on polyM of A_1_R_1_R_2_R_3_ (*F*_G_ = 0.72, *F*_GG_ = 0.61 after 23 h), but the enzyme concentration is not stated. The 2006 study saw no activity of A_1_R_1_R_2_R_3_ on polyMG, which differs from the observations of Ertesvåg (1998) and our observations of AlgE1 (+A_1_ −A_2_).

The AlgE1-derived A_1_R_1_R_2_R_3_ and A_2_R_4_ were, as discussed, the subject of several studies. Possibly, the activity of the A-module in these truncated enzymes would be different from the activity of each A-module in the full-length construct, for example, due to substrate binding. However, the study of AlgE1 with either A-module inactivated does not show any significant difference in activity or product formation compared to the two truncated enzymes. The activity of A_2_ is not impacted by the removal or inactivation of A_1_.

Though the substrate for A_1_ in AlgE1 WT must be MG, A_1_ alone or with an inactive A_2_ is barely active on polyMG. Possibly, R_4_ and most of A_2_ cannot bind polyMG, which might hinder the substrate reaching A_1_, which would also explain why AlgE1 WT has a similar low activity on polyMG. But low binding of A_2_R_4_ to polyM would not explain why A_1_ alone does not have higher activity on polyMG. It can therefore be concluded that A_1_ must need to be in a full-length construct with an active A_2_-module to be able to bind properly and process on the substrate.

This asymmetric dependency of A_1_ and A_2_ could be explained by A_2_ reacting with the substrate first. Thereby, A_2_ would always be able to perform its reactions, while A_1_ might be dependent on the preceding preparation of the substrate by A_2_ to react.

The active control has a lower initial reaction rate and a lower total amount of epimerization than AlgE1 WT. The only difference is the “GGR” insert in A_2_ of the active control, while the active sites were unchanged, which means that this change of the construct disrupts the enzyme’s ability to bind the substrate or its processivity. Possibly, the three amino acids are affecting the protein structure or alginate-binding sites.

In total, these observations bring forward the question, what happens if both modules are present and active in the enzyme but organized differently?

### 3.3 Switching Positions of the A-Modules Show the Importance of the Collaborative Effect

Our hypothesis is that the two A-modules of AlgE1 are working on one alginate chain at the same time in a collaborative manner. It is furthermore hypothesized that if the relative position of the A-modules is switched, the cooperative effect will be impacted, which could result in lower activity, less epimerization, and shorter G-blocks. Furthermore, changing the relative position of the A-modules in the enzyme could potentially also give clues about the directional movement of the enzyme on the substrate. To investigate this, two new variants of AlgE1 were made, where in one, the position of the A-modules was switched (AlgE1 MS), and in the other, A_2_R_4_ was moved from the C-terminal position to the N-terminal position (AlgE1 AR) (Figure 4A).

**Figure 4:**
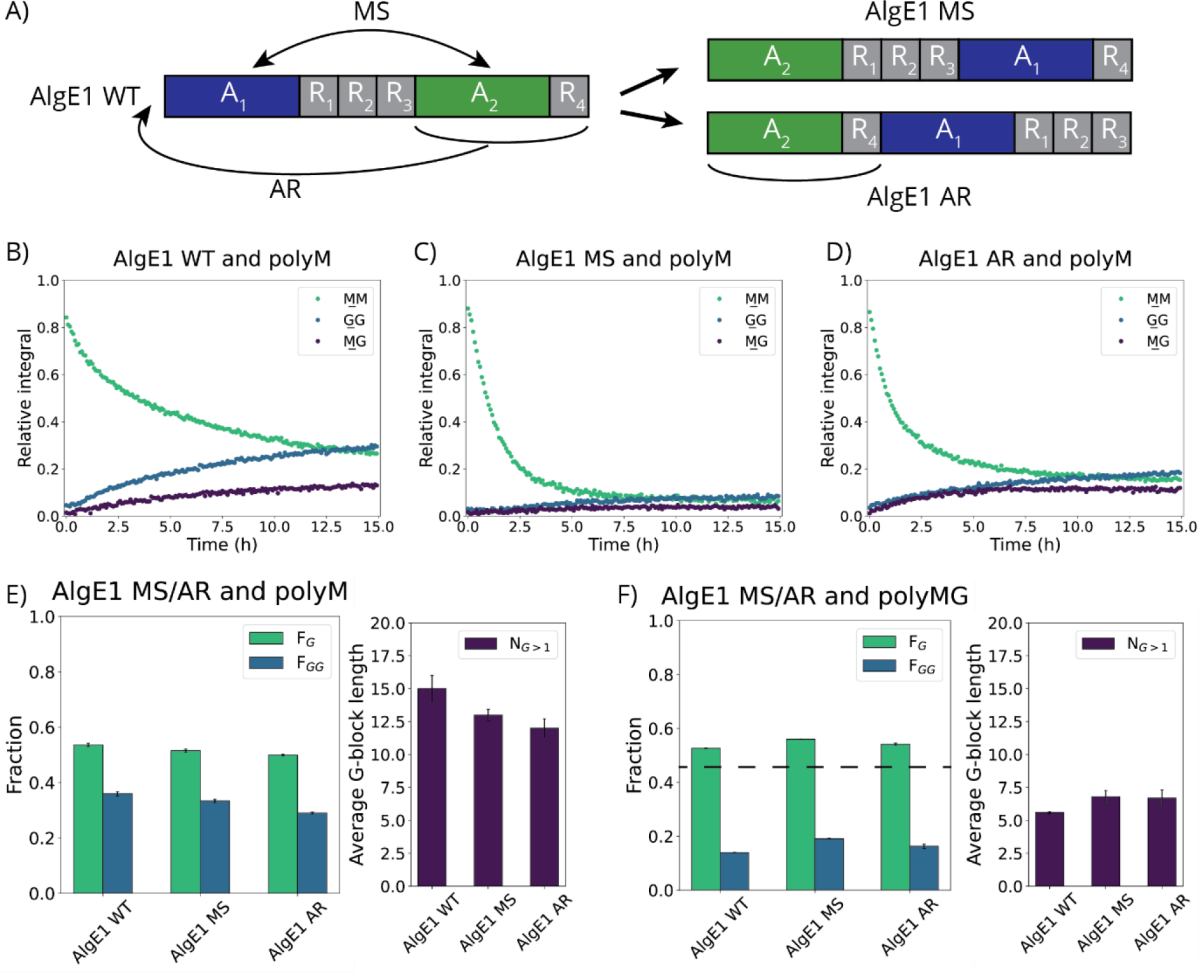
Reactions between polyM and AlgE1 WT, AlgE1 MS, and AlgE1 AR. A) Schematic overview representing AlgE1 WT and the rearrangements forming AlgE1 MS (top) and AlgE1 AR (bottom). B)–D) Normalized integrals from time-resolved NMR reaction with ^13^C-1-labeled polyM and AlgE1 WT (B), AlgE1 MS (C), or AlgE1 AR (D). The small GG signals in the reaction with AlgE1 MS and AR are due to the gel formation of the G-blocks. E) and F) NMR analyses of epimerization of polyM (E) or polyMG (F) after 48 h reaction (*n* = 3).

Total epimerization of polyM was measured after 48 h. The two new mutants were able to form G-blocks from polyM, i.e., they both reached high amounts of G (*F*_G_ = 0.50–0.52) and G-blocks of an average length of 12–13 consecutive G-residues (Figure 4 and Table S5). However, AlgE1 WT reached a slightly higher G-content (*F*_G_ = 0.54), and its G-blocks were 2–3 residues longer than those of AlgE1 MS and AlgE1 AR. This could be due to more gel formation in the reactions of AlgE1 MS and AR compared to AlgE1 WT (see below) as this would make the substrate inaccessible for the enzymes.

The reactions of ^13^C-1-labeled polyM and AlgE1 MS, AlgE1 AR, and AlgE1 WT were analyzed using time-resolved NMR spectroscopy. The initial rate of substrate disappearance was higher for the two mutants than for AlgE1 WT (Table 3), and less substrate remained after 15 h for both mutants compared to the WT, although the effect was largest for AlgE1 MS (Figure 4B–D). From the GG-signal in the spectra, it appears as if less GG-product is formed for AlgE1 MS and AlgE1 AR, even though more substrate has disappeared. This was, however, due to gel formation in the NMR tube where the generated consecutive G-residues had chelated Ca^2+^-ions present in the buffer, and since not being in solution, these residues will not appear in the NMR spectra. The gel was visible in the NMR tubes of AlgE1 MS and AlgE1 AR, but none could be observed in the tube of the AlgE1 WT reaction.

**Table 3:** Initial Reaction Rate of AlgE1 WT, AlgE1 MS, and AlgE1 AR with ^13^C-1-Labeled PolyM and Unlabeled PolyMG Based on Relative Integrals from Time-Resolved NMR Spectra

	PolyM MM consumption rate (mg/h)	PolyMG GG production rate (mg/h)
AlgE1 WT	0.393	0.018
AlgE1 MS	0.928	0.035
AlgE1 AR	0.845	0.031

Epimerization of polyMG was investigated, showing that both mutants and the WT were active. On polyMG, the two mutants created slightly more GG-product than AlgE1 WT, with AlgE1 MS creating the most (Figure 4F and Table S6). When monitoring reactions with polyMG (DP 70–90) using time-resolved NMR, the same trend was observed by following the formation of GG motifs. The initial reaction rate was fastest for AlgE1 MS and slowest for AlgE1 WT (Table 3).

Switching the A-modules of AlgE1 impacted the reaction rate, product formation, and processivity. This suggests that the A-modules in WT collaborate by simultaneously working on the same alginate chain. If the A-modules worked independently or less processively, their relative positions would not affect the activity. Therefore, in AlgE1 WT, the A-modules must act concurrently on the same chain, while in AlgE1 MS and AR, the two A-modules work independently of each other.

This theory is supported by the difference in the overall shape of AlgE1 in the absence and presence of the substrate. In Section 3.1, it was described how AlgE1 is more extended when the substrate is present, and the modeled conformations show that AlgE1 takes up a more linear shape. This change in shape to accommodate the substrate is in accordance with the cooperative binding of all modules of AlgE1.

AlgE1 WT has low activity on polyMG, which has also previously been reported.^28^ It was hypothesized that switching around the modules of AlgE1 WT could increase its polyMG activity, especially for AlgE1 AR, as that would place R_1_R_2_R_3_ terminally in the enzyme, since in AlgE1 WT, presumably R_1_R_2_R_3_ binds the MG product formed by A_2_. However, the results do not show a large increase in the MG activity for either mutant. Slightly more G-block is formed at a slightly higher rate than with the WT. AlgE1 AR has lower activity than AlgE1 MS. Thus, it cannot be concluded that the R_1_R_2_R_3_ modules have an impact on the MG activity of the enzyme.

Reaction of AlgE1 MS and AR resulted in gel formation as seen by the loss of the NMR signal. The formation of the gel could be due to the two mutants both forming shorter G-blocks than AlgE1 WT. Several shorter G-blocks result in several junction zones where gel formation can take place, while one single long G-block will not be able to form gel.^61^

This investigation highlights the significance of the relative position of the A-modules of AlgE1, and along with the A-module inactivation investigation, it indicates that the A-modules work simultaneously in collaboration on the alginate substrate. As A_2_ is active on MM forming MG and A_1_ is active on MG forming GG, it is reasonable to hypothesize that AlgE1 moves along the substrate with the C-terminal first. This hypothesis was tested using specific chimeras of AlgE1.

### 3.4 Chimeras of AlgE1 and AlgE7 Gives Insights into the Processivity of AlgE1

While AlgE1 has one function (epimerizing M-residues to G-residues), AlgE7 is bifunctional and harbors both epimerase and lyase activities.^24,34^ Replacing one of the two AlgE1 A-modules with the A-module of AlgE7 enables the investigation of the effect of introducing an A-module with lyase activity into AlgE1. By analyzing the reaction and product patterns of these chimeric enzymes, it is possible to gain an understanding of what end of the enzymes first reacts with the substrate.

Two chimeric enzymes were constructed where the AlgE7 A-module was inserted into AlgE1 replacing either A_1_ (AlgE 7–1) or A_2_ (AlgE 1–7) (Figure 5A). The reactions between the chimeras and the substrates polyM, polyMG, and polyG were investigated using an absorbance-based assay. Both enzymes displayed lyase and epimerase activity on polyM (Figure S8), while they showed primarily epimerase activity and only a low level of lyase activity on polyMG (Figure S9) and no activity on polyG (Figure S10). The reaction between the chimeras and ^13^C-1-labeled polyM was investigated using time-resolved NMR.

**Figure 5:**
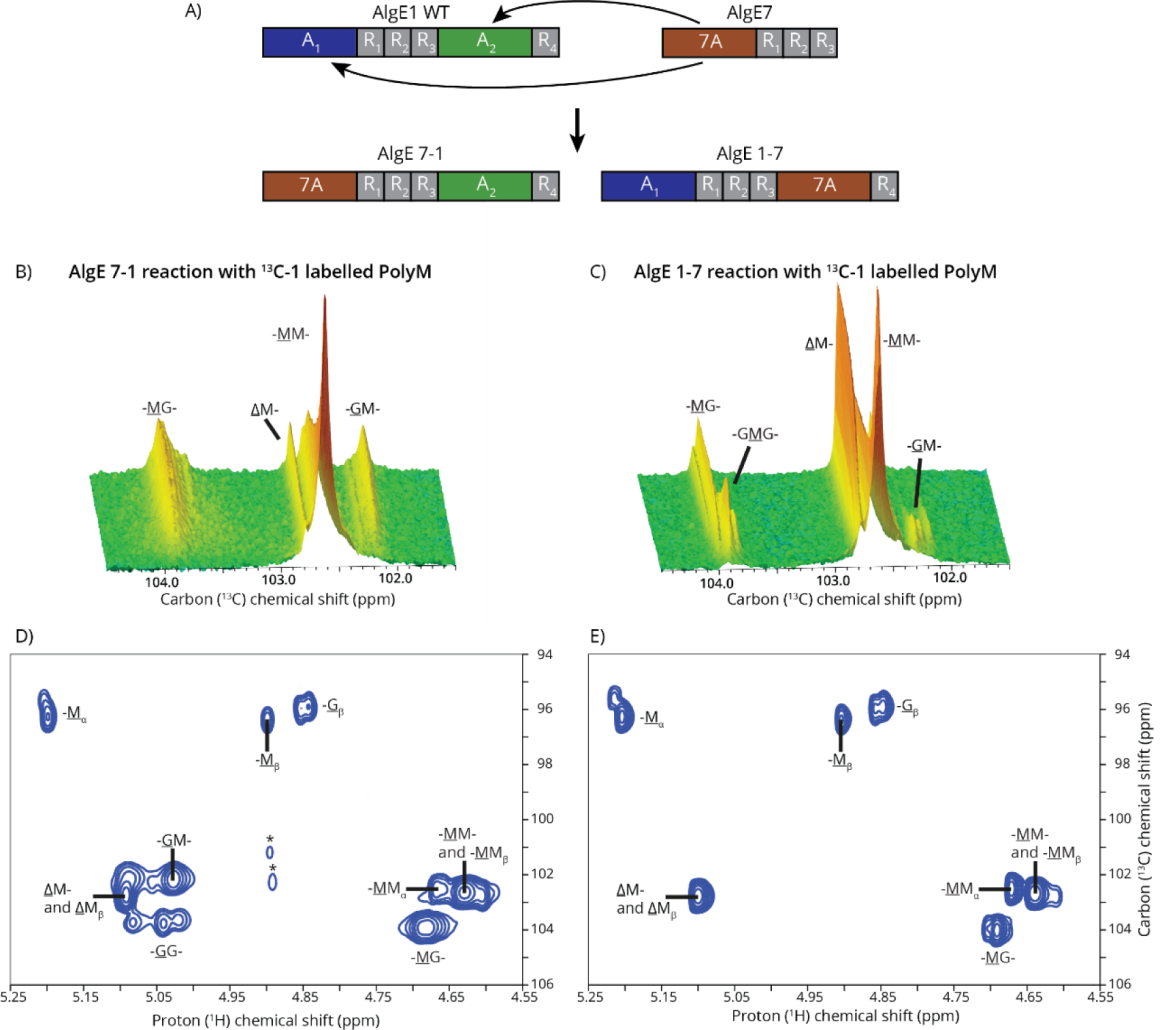
Reaction between the AlgE1 + AlgE7 chimeras. A) Schematic overview of the two chimeras formed by inserting the A-module of AlgE7 (7A) in place of the A-modules of AlgE1. B) and C) Reaction between ^13^C-1-labeled polyM and AlgE 7–1 (B) or AlgE 1–7 (C) observed using time-resolved NMR. Both epimerase and lyase reactions take place, forming MG-blocks and ΔM-containing products. D) and E) ^1^H–^13^C HSQC recorded after reaction between AlgE 7–1 (D) or AlgE 1–7 (E) and ^13^C-1-labeled polyM showing that both epimerization and lyase reaction take place. Substrate impurities are marked with “*”.

AlgE 1–7 has both epimerase and lyase activity on polyM. AlgE 1–7 forms MG-blocks and cleaves between M|M, M|G, and G|M, forming ΔM-containing lyase products (Figure 5). This is similar to the mode of action of AlgE7,^35^ suggesting that primarily the 7A module reacts with polyM. AlgE 1–7 is not able to generate G-blocks, which one would expect if the AlgE1 A_1_-module acted on the MG-stretches introduced by 7A. This suggests that the C-terminal 7A module cleaves the substrate directly after introducing GMG motifs such as AlgE7, which epimerizes and cleaves in the same binding event.^35^ This produces MG-blocks that are too short for A_1_ to bind and/or epimerize. On polyMG, the mode of action of AlgE 1–7 is more like AlgE1 (Figure S9), but a low level of lyase reactions also takes place. As both A_1_ and 7A can form G-blocks from polyMG, it is not surprising that AlgE 1–7 is active on polyMG forming G-blocks.

AlgE 7–1 reacts with polyM in a manner similar to that of AlgE1 by mainly forming MG- and GG-blocks, although AlgE 7–1 also has a small amount of lyase activity (Figure 5). None of the A-modules of AlgE 7–1 can alone form G-blocks directly from polyM. G-block formation can happen by both A-modules acting in succession on the same substrate chain, the C-terminal A_2_-module introducing MG-blocks, and afterward the N-terminal 7A mainly creating G-blocks from the MG-blocks formed by A_2_. The G-blocks could also be formed by the enzyme detaching and rebinding.

AlgE 7–1 has a much higher epimerase activity on polyMG than AlgE1. On polyMG, AlgE 7–1 reacts like AlgE7.

To determine whether the chimeric enzymes AlgE 1–7 and AlgE 7–1 behave in a processive manner, after reaction of the time-resolved NMR analysis, the samples were analyzed using HPAEC-PAD. On polyM, both enzymes cleave alginate into a few defined oligomers (Figure S11), whereas with polyMG, a range of products were created, and the analysis showed no indication of processivity (Figure S12). To further investigate the processivity on polyM, reactions between AlgE 7–1 and AlgE 1–7 with polyM were run for 24 h, with samples taken at specific time intervals and analyzed by HPAEC-PAD (Figures S13 and S14). The defined oligomers were already present after less than 1 min reaction for both enzymes and increased in intensity over time. This implies that the enzymes work processively as previously described for the AlgEs.^30–33^ Another possible explanation for why the short oligomeric products show up straight away could be that AlgE 7–1 and AlgE 1–7 have a binding preference and perform a preferred attack mechanism, instead of being processive.

AlgE 1–7 acts like AlgE7, meaning that the reaction performed by the 7A module takes place before the A_1_ reaction. AlgE 7–1 forms G-blocks, which can only happen if A_1_ works first and then 7A. Assuming that the two chimeras process in the same direction as AlgE1, AlgE1 must process along the substrate chain with the C-terminus first.

To further test this hypothesis and ensure that the difference between AlgE 7–1 and AlgE 1–7 is not due to the difference between A_1_ and A_2_, two new chimeric enzymes were made. Here, the relative positions of the two A-modules in AlgE 1–7 and AlgE 7–1 were switched to create two new enzymes: AlgE 1–7 MS and AlgE 7–1 MS (Figure 6A). These were also analyzed using time-resolved NMR (Figure 6). AlgE 1–7 MS and AlgE 7–1 MS show the same reaction pattern, and the same products are formed. Both form MG motifs and some GG motifs as well as a ΔM-containing lyase product (Figure 6).

**Figure 6:**
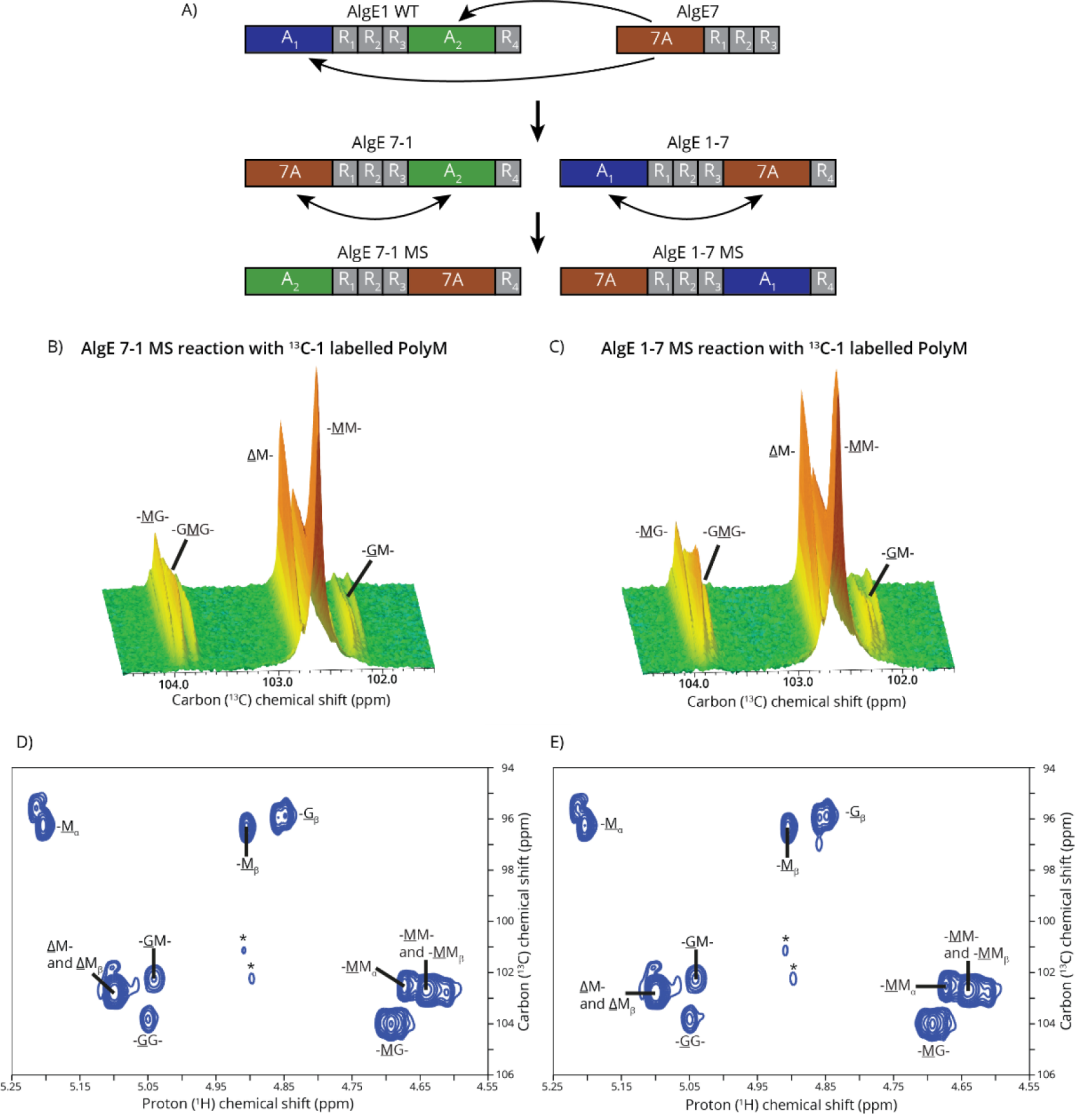
Reaction between AlgE1 + AlgE7 module-switch chimeras. A) Schematic overview of all four chimeras formed by inserting the A-module of AlgE7 (7A) in place of the A-modules of AlgE1 and then switching around the modules. B) and C) Reaction between ^13^C-1-labeled polyM and AlgE 7–1 MS (B) or AlgE 1–7 MS (C) observed using time-resolved NMR with key peaks labeled. Both epimerase and lyase reactions occur, forming MG-blocks and ΔM-containing products. D) and E) ^1^H–^13^C HSQC recorded after reaction between AlgE 7–1 MS (D) or AlgE 1–7 MS (E) and ^13^C-1-labeled polyM showing that both epimerization and lyase reaction take place. The primary products formed are lyase products and MG-blocks, and a small amount of GG-blocks is present as well. Substrate impurities are marked with “*”.

The only impact of switching the modules of AlgE 1–7 was that AlgE 1–7 MS formed a small number of G-blocks. A_1_ is not active on polyM, but A_1_ can form some G-blocks from polyMG (Section 3.2), so possibly A_1_ of AlgE 1–7 MS reacted with uncleaved stretches of MG formed by the 7A module of another enzyme unit.

When the modules of AlgE 7–1 were switched around to create AlgE 7–1 MS, the activity changed completely. AlgE 7–1 MS forms much fewer G-blocks than AlgE 7–1 and instead primarily forms lyase products. This loss of G-block-forming ability and the large increase in lyase activity could be due to the 7A module reacting before A_2_. In AlgE 7–1 MS, the 7A module is located at the C-terminus, further confirming that the chimeras process with the C-terminus first.

Further supporting the hypothesis that AlgE1 processes with the C-terminal first is that the C-terminal A_2_-module converts polyM to MG and the N-terminal module converts MG to G-blocks. If the two A-modules are to react on the same alginate chain simultaneously forming very long G-blocks, they would have to process with the C-terminal first.

It is possible that the direction of translocation could be impacted by changing the relative positions of the modules or introducing the 7A module. If the MS/AR mutants had a changed directionality compared to the WT, the reaction pattern would not have been impacted by the switching position of the modules. Rather, it seems that the MS/AR mutants might have switched from being processive to utilizing a preferred attack mechanism. If switching the order for the A-modules (e.g., for the MS chimeras) resulted in them moving in the opposite direction than the WT, then the reaction of AlgE 1–7 MS should have been the same as that of AlgE 1–7 as the order of the A-modules would not matter.

A study by Gaardløs et al. used molecular simulations to conclude that AlgE4 processes with the N-terminal first.^62^ Since all AlgE epimerases are structurally very similar, one might assume that all AlgEs process in the same manner. Nevertheless, our study and the 2021 study have reached different conclusions, possibly revealing the limitations of computational studies or indeed that AlgE1 and AlgE4 process with opposite ends in front.

Several studies have approached the question of the directional movement of epimerases on the alginate substrate. Two structures of AlgEs (the A-module of AlgE4 and the full length *Ac*AlgE3) with substrate bound have been published, both showing the C-terminal end of the AlgEs and the nonreducing end of alginate pointing in the same direction,^63,64^ which would mean that the AlgEs move toward the nonreducing end of the substrate. In a 2006 study, it was concluded that AlgE1 and AlgE6 epimerize the substrate from the nonreducing end toward the reducing end,^33^ thus reaching the opposite conclusion. A 2021 study using molecular modeling showed that AlgE4 processes with the N-terminal first, in the direction of the reducing end of the polyM chain. Because of occurring ambiguity among different works reported in the literature, it is currently not possible to conclude whether the epimerases move toward the reducing or nonreducing end of alginate.

## 4 Conclusion

This study provides new insights into the mode of action of AlgE1, particularly the interplay between its modules and its directionality as a mannuronate C-5 epimerase. SEC-SAXS analysis revealed that AlgE1 adopts an extended conformation in the presence of the polyM substrate, supporting a cooperative binding mechanism.

Inactivation studies confirmed that both A-modules are active in the full-length enzyme with A_1_ showing the highest activity when A_2_ was present and functional. Variants with repositioned A-modules exhibited altered reaction patterns, indicating that the modules work collaboratively on the same alginate chain. SAXS data further support this cooperative interaction.

Chimeric enzymes combining AlgE1 and AlgE7 modules demonstrated that AlgE1 processes alginate, with its C-terminal end moving first along the substrate, confirming its directional movement. These findings enhance our understanding of AlgE1’s processivity and epimerase function.

Given the industrial demand for G-rich alginates, understanding and optimizing epimerase function are crucial for producing tailored alginate products. This study contributes to the potential industrial applications of AlgE1 and related enzymes.

## Supplementary Material



## Notes

SEC-SAXS raw data and details on samples, collection and analysis, EOM models and their fit with the SAXS data, graphs of absorbance-based assays, HPAEC-PAD chromatograms, and tables of results of endpoint NMR analyses (PDF)

## Abbreviations

CV: column volume
Δ: 4-*deoxy*-l-*erythro*-hex-4-enopyranosyluronate
DP: degree of polymerization
EDTA: ethylenediaminetetraacetic acid
G: α-l-guluronic acid
HPAEC-PAD: high-performance anionic exchange chromatography coupled with pulsed amperometric detection
HSQC: heteronuclear single quantum coherence
IPTG: isopropyl β-d-1-thiogalactopyranoside
LB: Luria Broth
M: β-d-mannuronic acid
NMR: nuclear magnetic resonance
TSP: 3-(trimethylsilyl)-propionic-2,2,3,3-d_4_ acid sodium salt
UPW: ultrapure water
