# Supplementary material for: Mode of Action of AlgE1: A Modular Mannuronate C‑5 Epimerase
Source: Biochemistry. 2025 Jun 23;64(14):3030–44. doi: 10.1021/acs.biochem.5c00156 (PMC12269086; doi:10.1021/acs.biochem.5c00156)
Supplement: Supplementary file 1 [file bi5c00156_si_001.pdf]

Supporting Information
Mode of Action of AlgE1: A Modular Mannuronate C-5 Epimerase

*Agnes B. Petersen[1], Anita Solem[1], Gerd Inger Sætrom[1], Håvard Sletta[2], Mirjam Czjzek[3], Finn L. Aachmann[1], Anne Tøndervik[2,]**

[1] Norwegian Biopolymer Laboratory (NOBIPOL), Department of Biotechnology and Food Science, NTNU Norwegian University of Science and Technology. 7034 Trondheim, Norway.

[2] Department of Biotechnology and Nanomedicine, SINTEF Industry. 7034 Trondheim, Norway.

[3]Station Biologique de Roscoff, Sorbonne Université, CNRS, Laboratoire de Biologie Intégrative des Modèles Marins, LBI2M. 29680 Roscoff, France.

Table of Contents

Supplementary figures:

Figure S1. SAXS quality assessment of experimental data.
Figure S2. SEC-SAXS data.
Figure S3. Solution SAXS curves of AlgE1 without substrate and with oligoM DP20, and structural interpretation of the experimental data.
Figure S4. Solution SAXS curves of AlgE1 in presence of DP20 substrate and structural interpretation of the experimental data.
Figure S5. EOM calculations of Rg distributions for the statistical pool and AlgE1 & AlgE1-DP20.
Figure S6. Platereader assay of AlgE1 mutants' reaction with polyM.
Figure S7. Reaction between AlgE1 (+A1 -A2) and polyM.
Figure S8. Platereader assay of reaction between AlgE1/7 chimers and polyM.
Figure S9. Figure S9: AlgE1 + AlgE7 chimers reaction with polyMG.
Figure S10. Platereader assay of reaction between AlgE1/7 chimers and polyG.
Figure S11. HPAEC-PAD spectra recorded of end of reaction with AlgE 7-1 and AlgE 1-7 with ^{13}C-1 labelled polyM.
Figure S12. HPAEC-PAD spectra recorded of end of reaction with AlgE 7-1 and AlgE 1-7 with polyMG.
Figure S13. HPAEC-PAD spectra recorded of reaction of AlgE 1-7 with polyM.
Figure S14. HPAEC-PAD spectra recorded of reaction of AlgE 7-1 with polyM.

Supplementary Tables:

Table S1. Primers used in PCR-reactions.
Table S2. Details on SEC-SAXS samples, data collection, analysis, and 3D modelling details.
Table S3. Results of endpoint NMR measurements after 48 h of the reactions of the four inactivated mutants and AlgE1 WT with polyM.
Table S4. Results of endpoint NMR measurements after 48 h of the reactions of the four inactivated mutants and AlgE1 WT with polyMG.
Table S5. Results of endpoint NMR measurements after 48 h of the reactions of the module-switch mutants AlgE1 MS and AlgE1 AR, and AlgE1 WT with polyM.
Table S6. Results of endpoint NMR measurements after 48 h of the reactions of the module-switch mutants AlgE1 MS and AlgE1 AR, and AlgE1 WT with polyMG.

Supplementary Figures

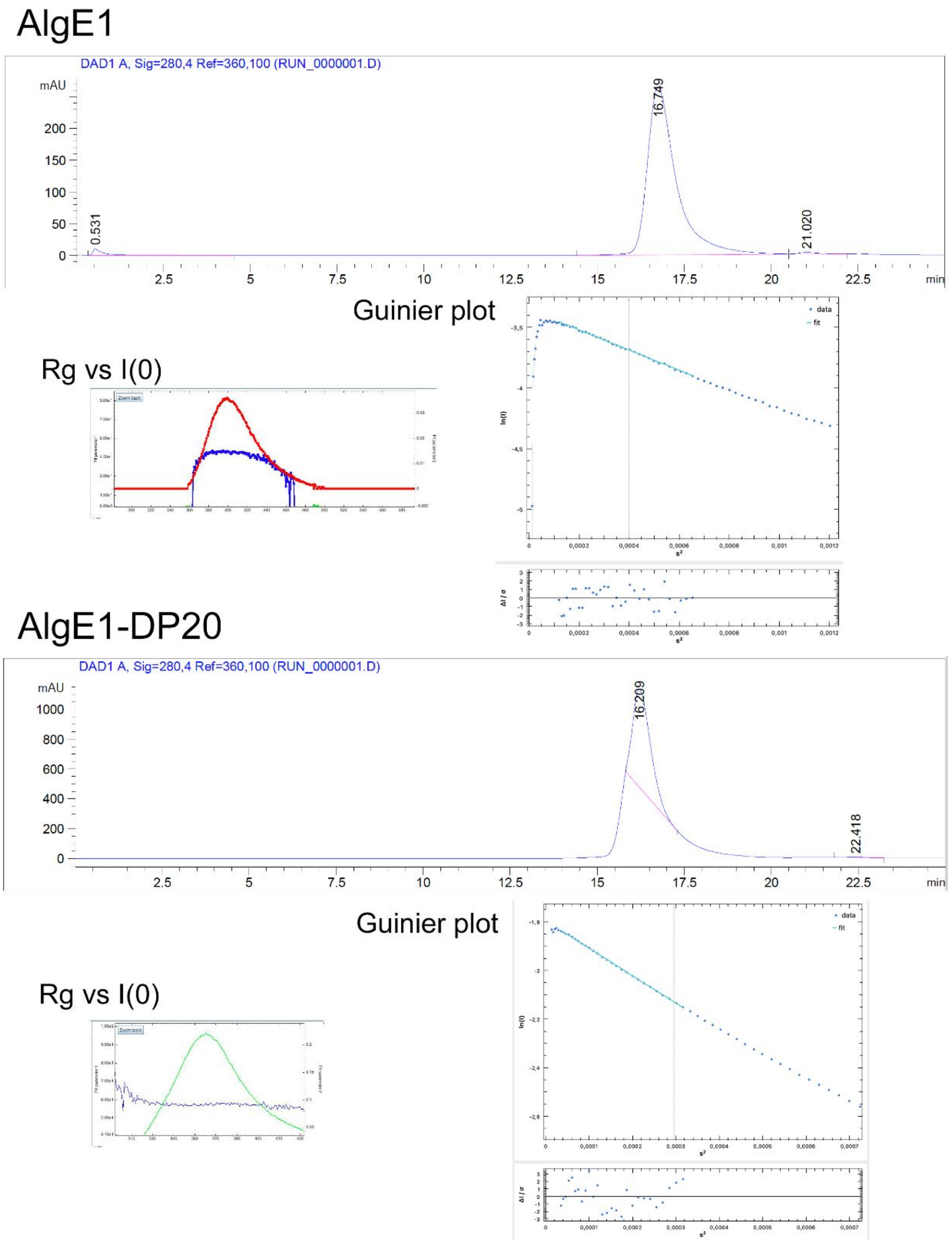

Figure S1 | SAXS quality assessment of experimental data on AlgE1 and AlgE1-DP20. The different panels show: the raw data for the SEC elution-profiles, the Rg versus Intensity plots and the linearity of the Guinier-plots.

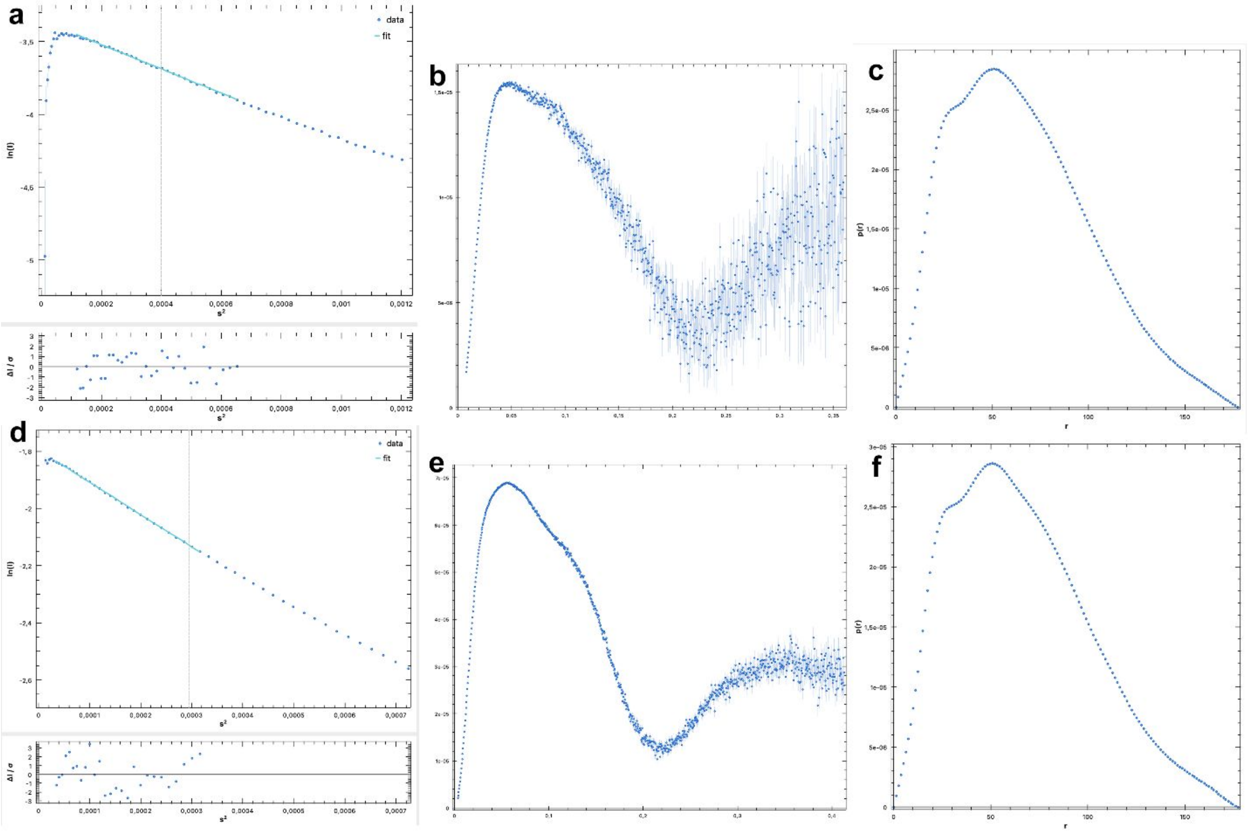

Figure S2 | SEC-SAXS data. The experimental SEC-SAXS data were processed and evaluated for **a-c** AlgE1 and **d-f** AlgE1-DP20. **a+d** Linear Guinier region of the experimental data. The light-blue, straight line represents the Guinier approximation. The residual errors are indicated in the boxes underneath the curves. **b+e** Normalized Kratky representation. **c+f** Distance distribution function, *p(r)*.

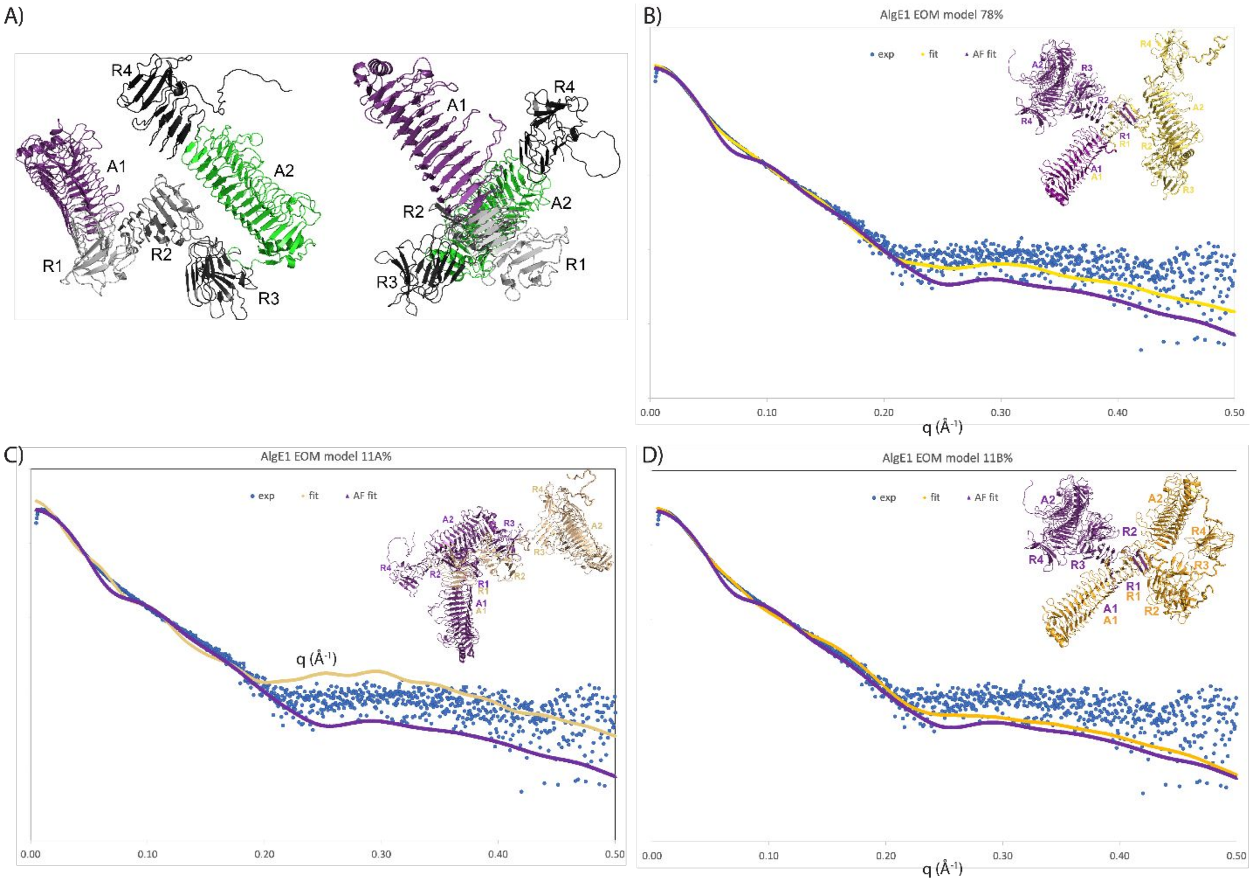

Figure S3 | Solution SAXS curves of AlgE1 without substrate and with oligoM DP20, and structural interpretation of the experimental data. **A)** Overall structural model of AlgE1 as calculated by Alphafold3 (AF3) shown in two perpendicular orientations. The individual domains are labelled from A1 to R4. **B) C), and D)** The structural representation of three major, individual structural models as calculated by EOM, that together best fit the experimental curve (shown in Figure 2C), are shown as inset (yellow models) and are super-imposed to the AF3 model (purple models). The corresponding fitted curves to the individual models are shown in yellow (C χ^2 of 9.87; D χ^2 of 9.84, E χ^2 of 7.87), while the fit of the AF3 model are shown as purple curves χ^2 of 7.19). The blue dots represent the experimental data.

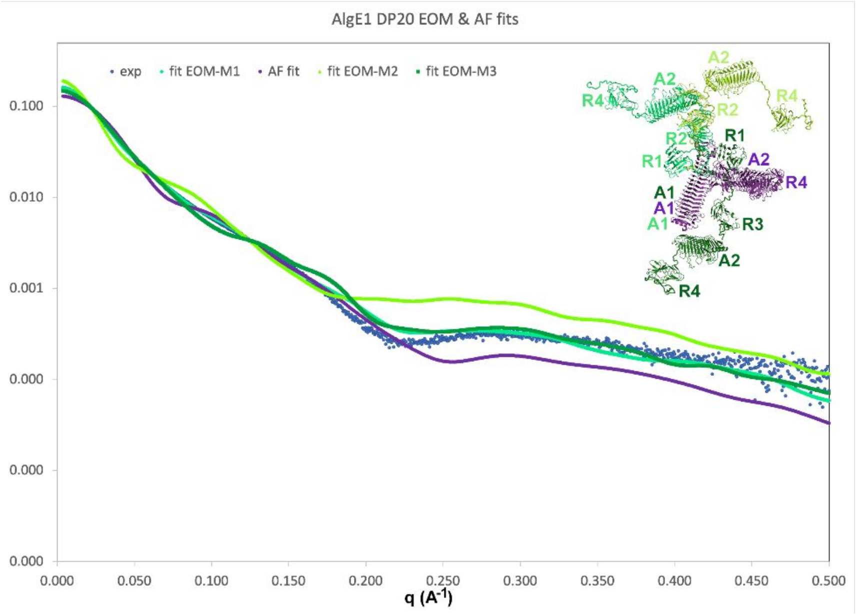

Figure S4 | Solution SAXS curves of AlgE1 in presence of DP20 substrate and structural interpretation of the experimental data. The structural representation of three major, individual structural models (lime-green, blue-green and green) as calculated by EOM, and superimposed onto the AF3 model (purple). The corresponding fitted curves to the individual models are shown represented with the same colours all give high χ^2 values (> 70), again the fit of the AF3 model is shown as a purple curve.

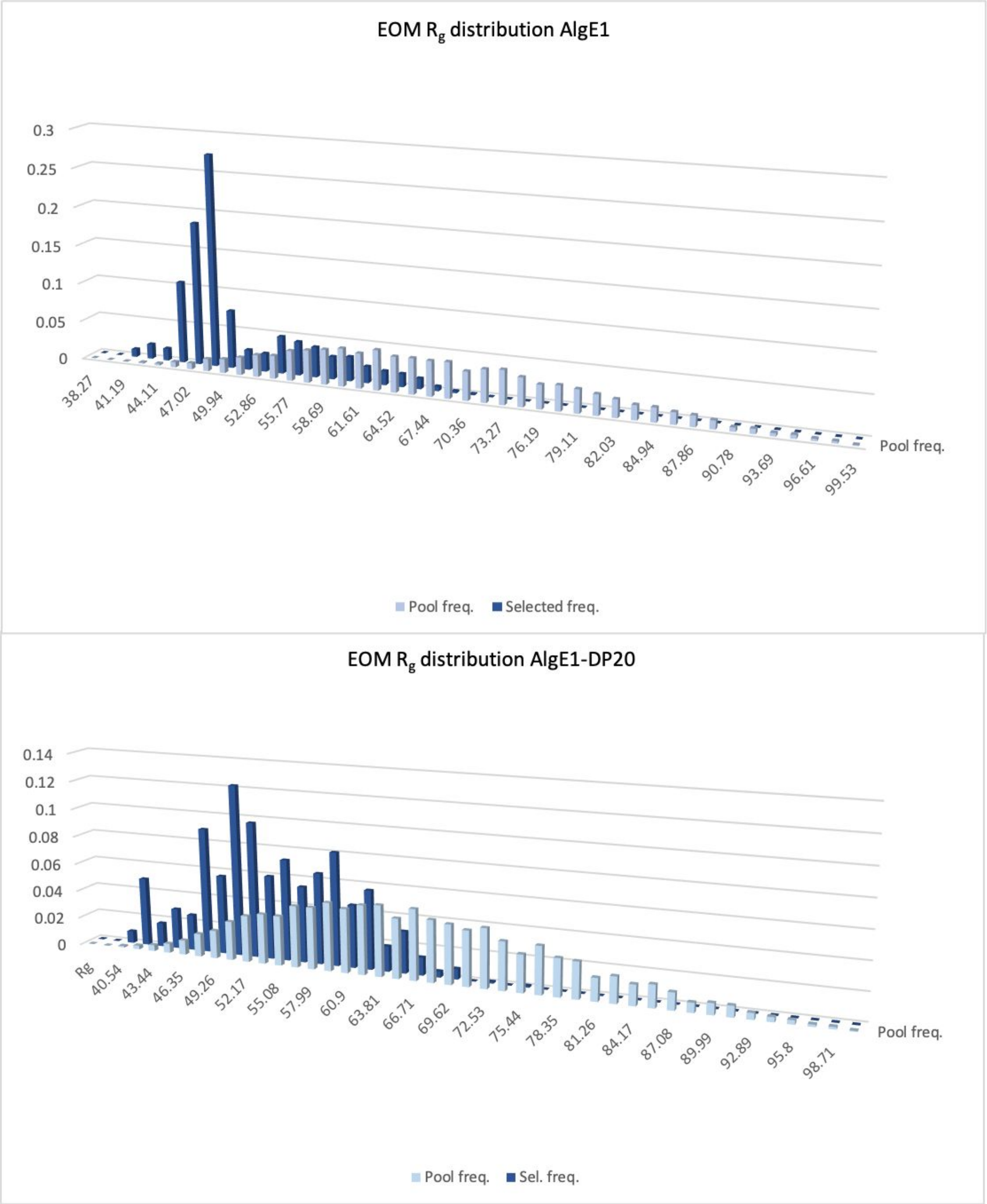

Figure S5 | EOM calculations of R_g distributions for the statistical pool (light blue) and AlgE1 & AlgE1-DP20 dark-blue. Top) The distribution shows the presence of two populations for AlgE1, a major, more compact one with average R_g of 50 Å and a second minor population with average Rg of 56 Å. Bottom) the presence of two populations is less marked, but as for AlgE1 the conformations are more compact than for the statistical pool.

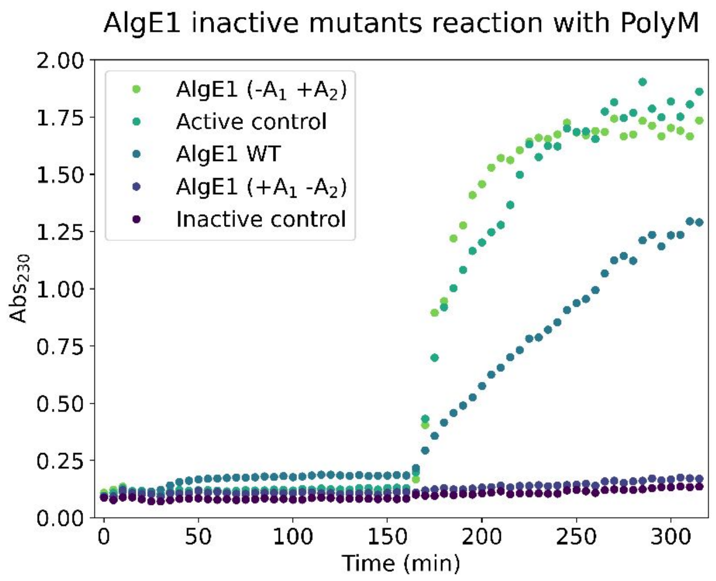

Figure S6 | Platereader assay of AlgE1 mutants reaction with polyM. After 150 min a G-specific lyase was added to each well. Thus the signal from 150-300 min corresponds to the epimerised Gs. (n=3)

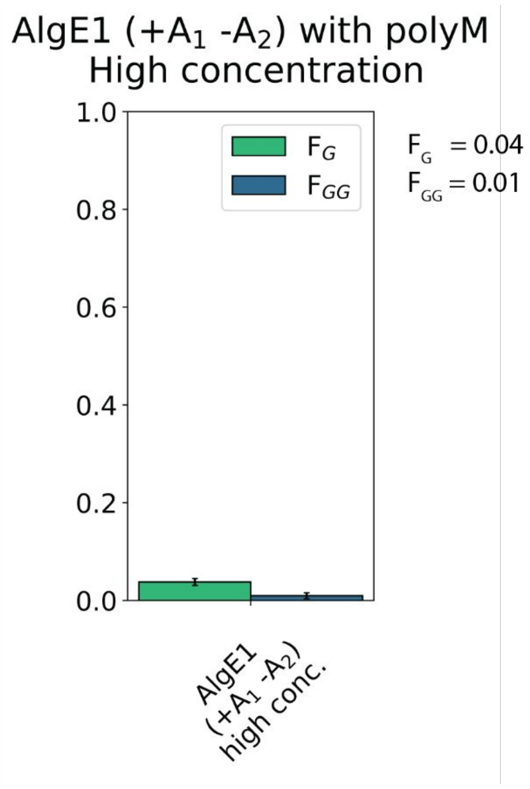

Figure S7 | Reaction between AlgE1 (+A_1 -A_2) and polyM at an enzyme concentration 5 times as high as for the other epimerisation reactions (125 µg/mL). With this high enzyme concentration, a small amount of G and GG product is formed. (n=3)

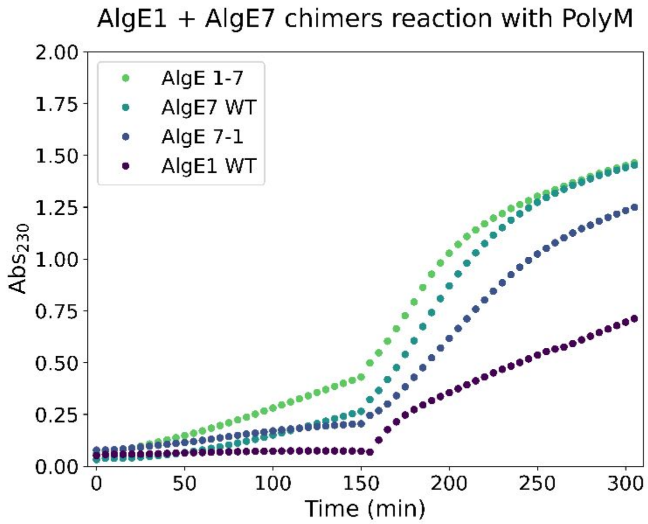

Figure S8 | Platereader assay of reaction between AlgE1/7 chimers and polyM. After 150 min a G-specific lyase was added to each well. Thus the signal from 150-300 min corresponds to the epimerised Gs. (n=3)

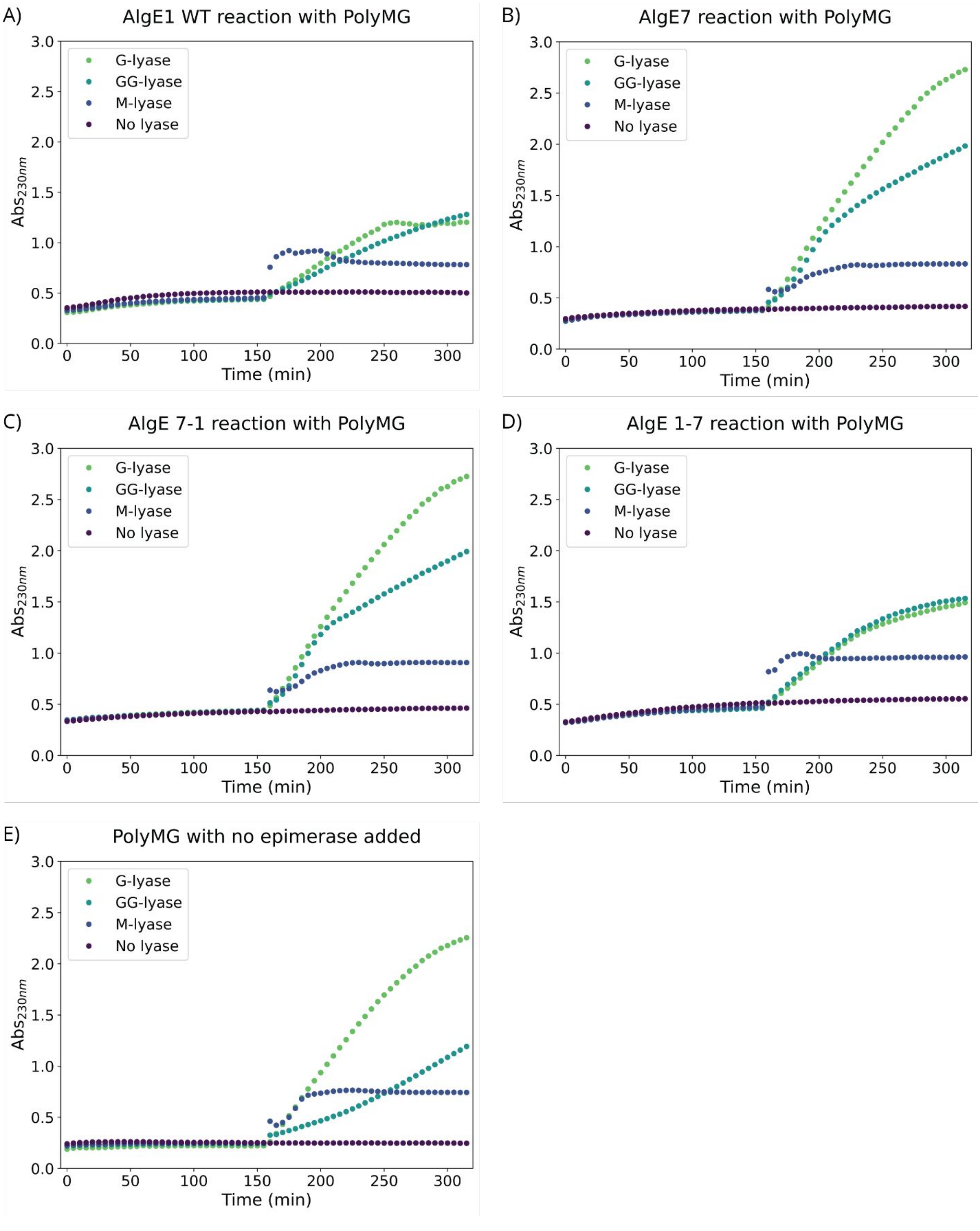

Figure S9 | AlgE1 + AlgE7 chimers reaction with polyMG. After 150 min either a G-specific, G-G specific, or M-specific lyase was added to the reaction. (n=3)

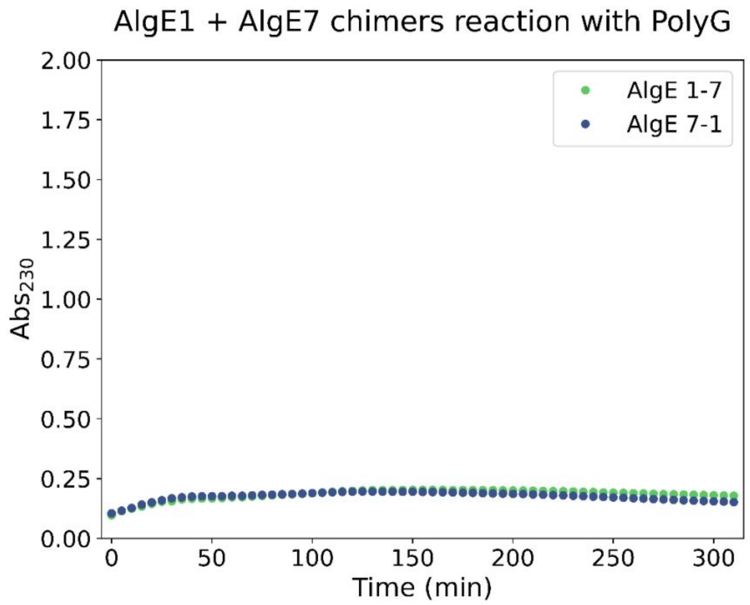

Figure S10 | Platereader assay of reaction between AlgE1/7 chimers and polyG. (n=3)

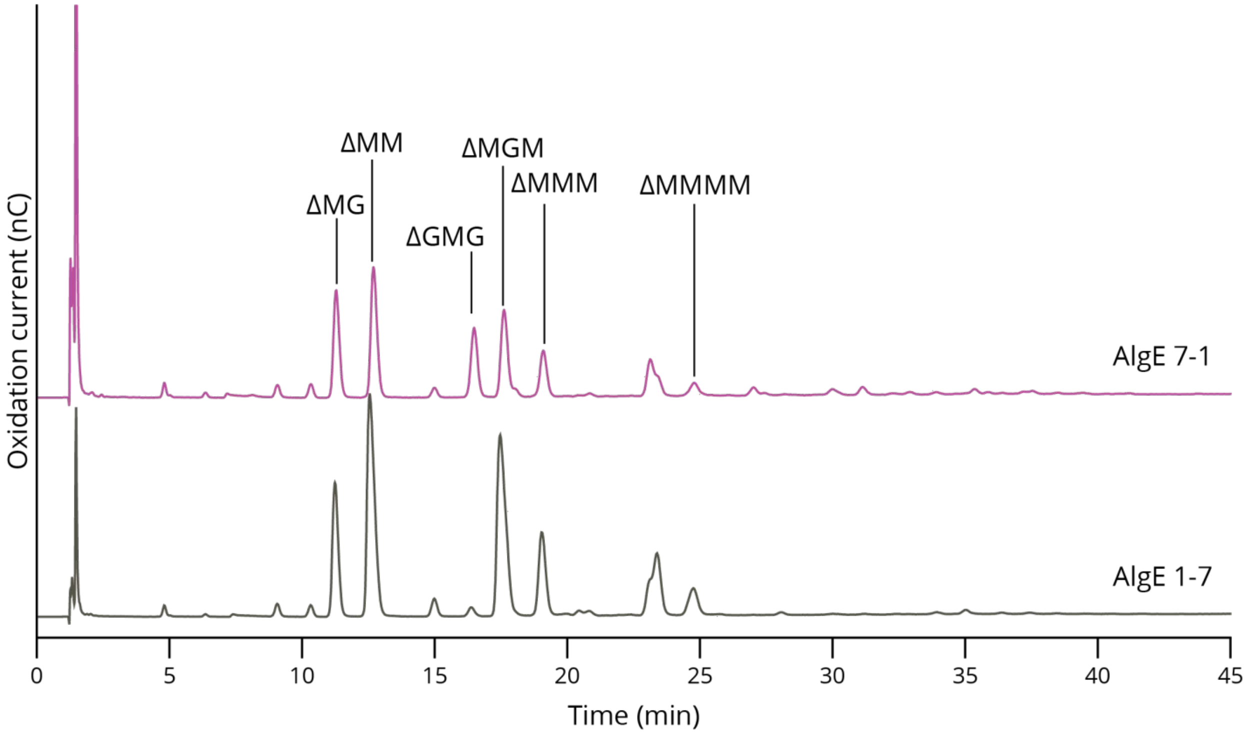

Figure S11 | HPAEC-PAD spectra recorded of end of reaction with AlgE 7-1 and AlgE 1-7 with ^{13}C-1 labelled polyM. Reaction was run in an NMR tube for 24 h, then the reaction mixture was extracted and diluted with UPW to a carbohydrate concentration of 0.3 mg/mL.

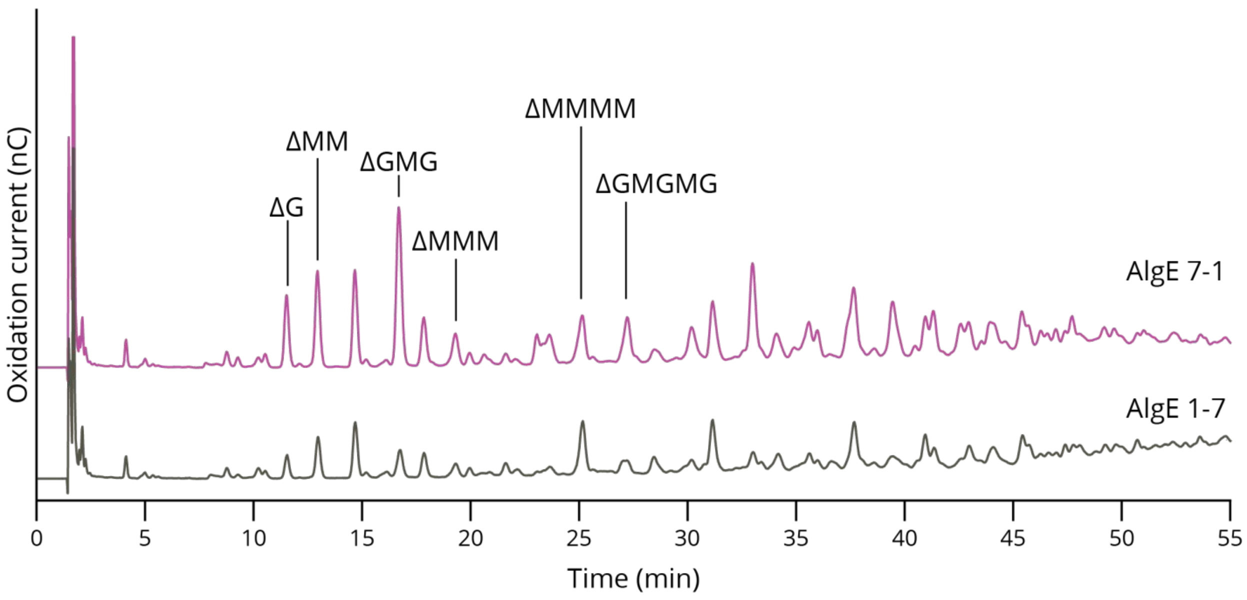

Figure S12 | HPAEC-PAD spectra recorded of end of reaction with AlgE 7-1 and AlgE 1-7 with polyMG. Reaction was run in an NMR tube for 24 h, then the reaction mixture was extracted and diluted with UPW to a carbohydrate concentration of 0.3 mg/mL.

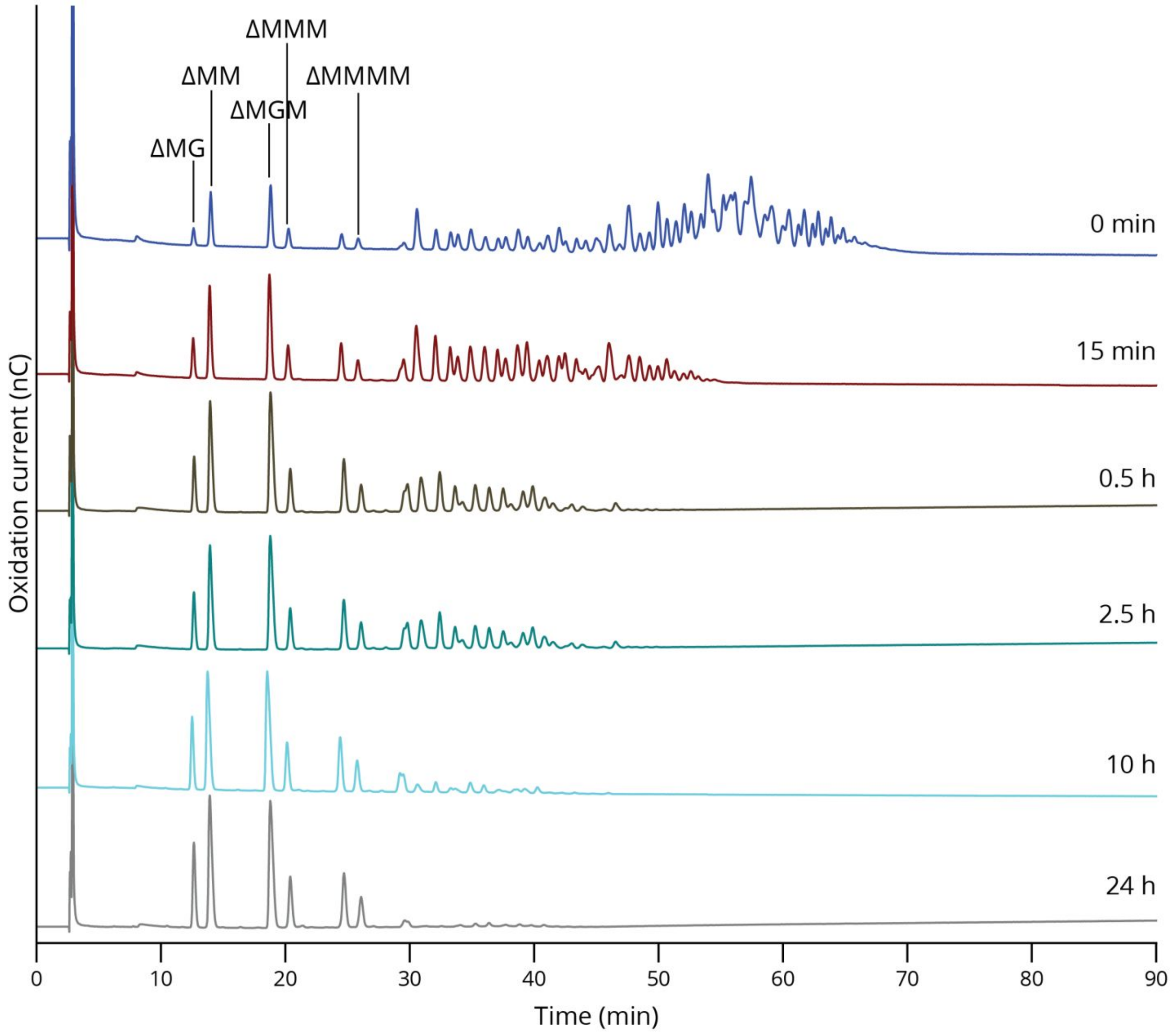

Figure S13 | HPAEC-PAD spectra recorded of reaction of AlgE 1-7 with polyM. Reaction was run in a plate reader for up to 24 h, with samples analysed at specific intervals.

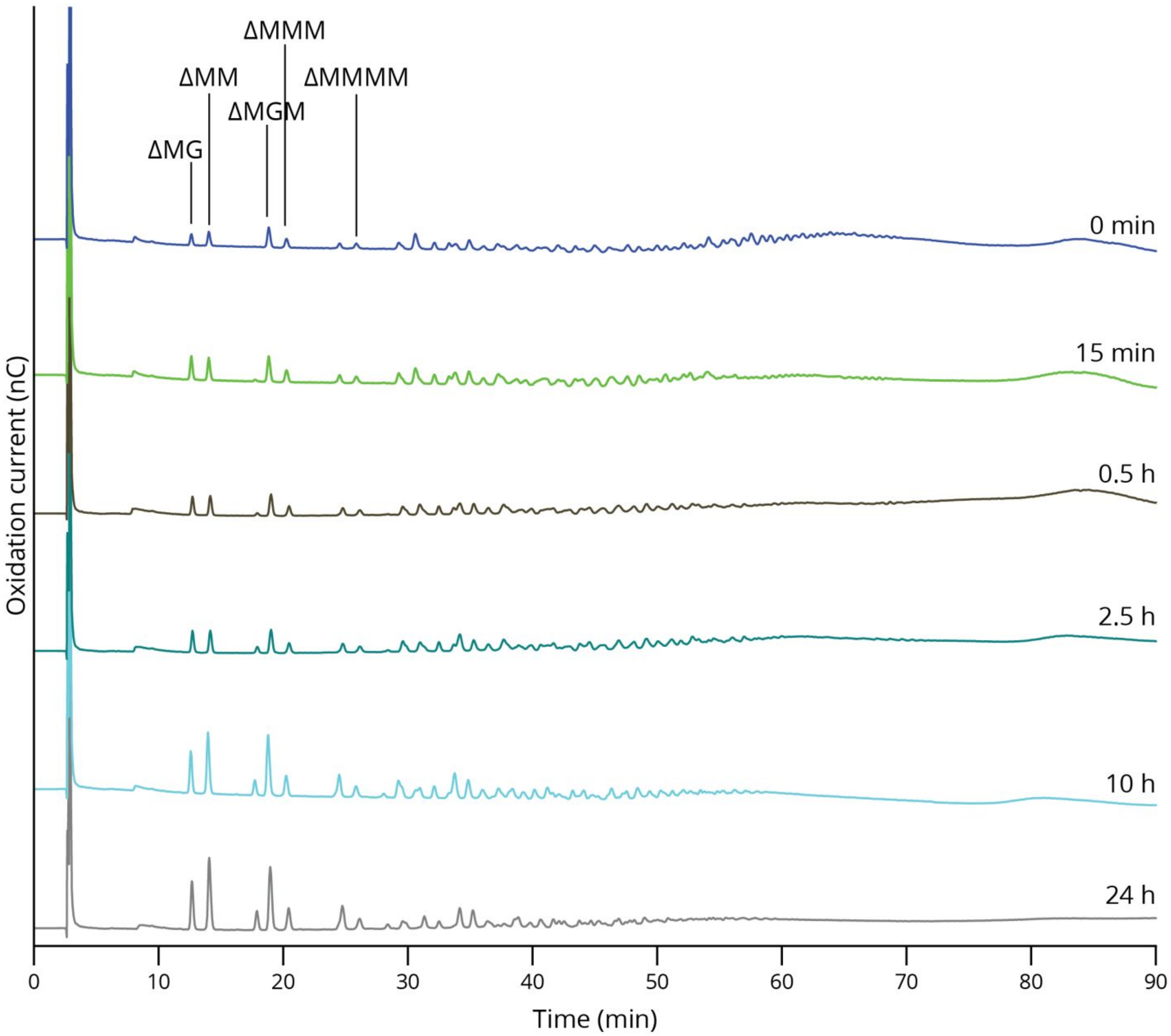

Figure S14 | HPAEC-PAD spectra recorded of reaction of AlgE 7-1 with polyM. Reaction was run in a plate reader for up to 24 h, with samples analysed at specific intervals.

Supplementary Tables

Table S1 | Primers used in PCR-reactions for the construction of chimeric enzymes and their respective application, target template, and sequence. All primers were sourced from Thermo Fisher Scientific (Waltham, USA).

Application	Template	Primer	Sequence (5'-3')
Construction of AlgE 1-7	pTrc99a-*AlgE1*	pTYB1_AlgE1_fwd	GGGCGATCGCAGGGAACCGACGGCA
		pTYB1_AlgE1_rev	CTTAACGTTGAACACGGCGGGCTCGAAG
	AlgE7A	AlgE7_A_fwd2	CCGCAGGGAACCGACGGCAAC
		AlgE7_A_rev2	GAACACGGCGGGCTCGAAGA
Construction of AlgE 1-7 MS	pTrc99a-*AlgE1*	AlgE17ms_vec_fwd	GAAGTGCCGCAGGGGACC
		AlgE17ms_vec_rev	CACGGCGGGCTCGAAGATC
	AlgE1 A1	AlgE17ms_ins_fwd	TGATCTTCGAGCCCGCCGTGAACGTCAAGGATTTCGGAGC
		AlgE17ms_ins_rev	TCGGTCCCCTGCGGCACTTCGCCGGACTGGCTGGAAAC
Construction of AlgE 7-1 MS	pTYB1-*AlgE 1-7*	AlgE71ms_vec_fwd	CAGTCCGGCAGTGGCCAG
		AlgE71ms_vec_rev	GACGTTGTAATCCATATGTATATCTCCTTCTTAAAG
	AlgE1	AlgE71ms_ins_fwd	TACATATGGATTACAACGTCAAGGACTTCGGCGCGCTG
		AlgE71ms_ins_rev	TGCTGGCCACTGCCGGACTGGCCGCCGGAAAGACTCGAATG

Table S2 | Details on SEC-SAXS samples, data collection, analysis, and 3D modelling details for AlgE1 and AlgE1 with oligoM DP20.

(a) Sample details.	**AlgE1**	**AlgE1+DP20**
Origin	*Azotobacter vinelandii*<br>UniProt Q44494	*Azotobacter vinelandii*<br>UniProt Q44494<br>+poly-M DP19-21
M_w from chem. comp. (aa)	147169.01 Da (1403aa)	same + poly-M 3862 Da
Sample environment	20 mM HEPES pH 6.9<br>75 mM NaCl, 5 mM $CaCl_2$	20 mM HEPES pH 6.9<br>75 mM NaCl, 5 mM $CaCl_2$
Sample temperature (°C)	15	15
In-beam sample cell, flow	1 mm quartz capillary, 0.3 ml/min	1 mm quartz capillary, 0.3 ml/min
Sample concentration(s) (mg/ml)	7	7

(b) SAXS data collection.
Data-acquisition/reduction software: FOXTROT 3.5.10-3979
Source/instrument description: SOLEIL SWING beamline
Measured q-range (qmin–qmax) ($Å^{-1}$): 0.0036–0.553
Method for scaling intensities: Absolute scaling (cm^{-1}) referenced to water
Exposure time(s), No. of exposures 990ms x 880 frames (sample); 990ms x 180 frames (buffer)

(c) SAXS-derived structural parameters.
Method(s)/software: PRIMUS, AUTORG and GNOM (ATSAS 3.1.3 (2)).

Guinier analysis	**AlgE1**	**AlgE1+DP20**
I(0) (cm^{-1})	0.032 ± 0.002†	0.17 ± 0.002†
Rg (Å)	49.79 ± 0.14	58.2 ± 0.09
qRg range (datapoint range)	0.64–1.21 (20–46)	0.35–1.04 (6–32)
Linear fit assessment (AUTORG fidelity)	0.17	0.00
PDDF/P(r) analysis		
I(0) (cm^{-1})	0.032 ± 0.0005	0.167 ± 0.004
Rg (Å)	52.6 ± 0.11	60.2 ± 0.4
Dmax (Å)	177.2	220.0
q-range ($Å^{-1}$)	0.012–0.3676	0.0078–0.390
P(r) reciprocal-space fit/CorMap P-value	1.12, 0.792	1.44, 0.71

(d) Scattering particle size.
Method(s)/software PRIMUS (ATSAS 3.1.3 (2); equation 1 in (3) for M from I(0)/c)

	**AlgE1**	**AlgE1+DP20**
Volume estimates (Å^3)	118750	146800
Porod volume VP (ratio to M)	124118 (0.83)	151554 (1.003)
M from I(0)/c	109804 (0.74)	149500 (0.99)

(e) Modelling.
Methods/software Dummy-atom (GASBORi), and the Ensemble Optimization Method (EOM) (5)

Shape modelling/software	DAMMif	DAMMif
q-range for fit (Å^{-1})	0.012–0.50	0.0126–0.50
Symmetry/anisotropy assumptions	P1	P1
Iterative closest point (average)	65.2	90.2
No. of individual model reconstructions	10	10

(f) Data and model deposition.	**AlgE1**	**AlgE1+DP20**
SASBDB ID	SASDxxx1	SASDyyy2

Table S3 | Results of endpoint NMR measurements after 48 h of the reactions of the four inactivated mutants and AlgE1 WT with polyM

Sample	F_G	F_M	F_{GG}	$F_{GM}=F_{MG}$	F_{MM}	$F_{GGM}=F_{MMG}$	F_{MGM}	F_{GGG}	$N_{G>1}$
AlgE1 WT	0.57	0.43	0.40	0.17	0.26	0.02	0.15	0.36	18
AlgE1 (+A$_1$ -A$_2$)	0.00	1.00	0.00	0.00	1.00	0.00	0.00	0.00	0
AlgE1 (-A$_1$ +A$_2$)	0.20	0.80	0.00	0.20	0.60	0.00	0.20	0.00	0
Active control	0.41	0.59	0.33	0.08	0.52	0.02	0.06	0.30	17
Inactive control	0.00	1.00	0.00	0.00	1.00	0.00	0.00	0.00	0

Table S4 | Results of endpoint NMR measurements after 48 h of the reactions of the four inactivated mutants and AlgE1 WT with polyMG

Sample	F_G	F_M	F_{GG}	$F_{GM}=F_{MG}$	F_{MM}	$F_{GGM}=F_{MMG}$	F_{MGM}	F_{GGG}	$N_{G>1}$
AlgE1 WT	0.53	0.47	0.14	0.39	0.09	0.03	0.36	0.11	6
AlgE1 (+A$_1$ -A$_2$)	0.51	0.49	0.11	0.40	0.09	0.03	0.37	0.07	5
AlgE1 (-A$_1$ +A$_2$)	0.45	0.55	0.00	0.45	0.10	0.02	0.43	0.00	1
Active control	0.51	0.49	0.10	0.40	0.09	0.03	0.38	0.07	5
Inactive control	0.45	0.55	0.00	0.45	0.10	0.02	0.43	0.00	1

Table S5 | Results of endpoint NMR measurements after 48 h of the reactions of the module-switch mutants AlgE1 MS and AlgE1 AR, and AlgE1 WT with polyM

Sample	F_G	F_M	F_{GG}	$F_{GM}=F_{MG}$	F_{MM}	$F_{GGM}=F_{MMG}$	F_{MGM}	F_{GGG}	$N_{G>1}$
AlgE1 WT	0.54	0.46	0.36	0.18	0.29	0.03	0.15	0.33	15
AlgE1 MS	0.52	0.49	0.33	0.18	0.30	0.03	0.15	0.30	13
AlgE1 AR	0.50	0.50	0.29	0.21	0.29	0.03	0.18	0.26	12

Table S6 | Results of endpoint NMR measurements after 48 h of the reactions of the module-switch mutants AlgE1 MS and AlgE1 AR, and AlgE1 WT with polyMG

Sample	F_G	F_M	F_{GG}	$F_{GM}=F_{MG}$	F_{MM}	$F_{GGM}=F_{MMG}$	F_{MGM}	F_{GGG}	$N_{G>1}$
AlgE1 WT	0.53	0.47	0.14	0.39	0.09	0.03	0.36	0.11	6
AlgE1 MS	0.56	0.44	0.19	0.37	0.07	0.03	0.34	0.15	7
AlgE1 AR	0.54	0.46	0.16	0.38	0.08	0.03	0.35	0.13	7
